# Comparative genomics profiling revealed multi-stress responsive roles of the CC-NBS-LRR genes in three mango cultivars

**DOI:** 10.3389/fpls.2023.1285547

**Published:** 2023-10-30

**Authors:** Muhammad Tahir ul Qamar, Muhammad Sadaqat, Xi-Tong Zhu, Huan Li, Xing Huang, Kinza Fatima, Mashal M. Almutairi, Ling-Ling Chen

**Affiliations:** ^1^ State Key Laboratory for Conservation and Utilization of Subtropical Agro-bioresources, College of Life Science and Technology, Guangxi University, Nanning, Guangxi, China; ^2^ Department of Bioinformatics and Biotechnology, Government College University Faisalabad (GCUF), Faisalabad, Pakistan; ^3^ Key Laboratory of Biology and Genetic Improvement of Oil Crops, Ministry of Agriculture, Oil Crops Research Institute, Chinese Academy of Agricultural Sciences, Wuhan, China; ^4^ Environment and Plant Protection Institute, Chinese Academy of Tropical Agricultural Sciences, Haikou, China; ^5^ Department of Pharmacology and Toxicology, College of Pharmacy, King Saud University, Riyadh, Saudi Arabia

**Keywords:** draft pan-genome, mango, NBS-LRR, multi omics, machine learning

## Abstract

The nucleotide-binding site-leucine-rich repeat (NBS–LRR) gene family is the largest group of disease resistance (R) genes in plants and is active in response to viruses, bacteria, and fungi usually involved in effector-triggered immunity (ETI). Pangenome-wide studies allow researchers to analyze the genetic diversity of multiple species or their members simultaneously, providing a comprehensive understanding of the evolutionary relationships and diversity present among them. The draft pan-genome of three *Mangifera indica* cultivars (*Alphonso, Hong Xiang Ya*, and *Tommy atkins*) was constructed and Presence/absence variants (PAVs) were filtered through the ppsPCP pipeline. As a result, 2823 genes and 5907 PAVs from *H. Xiang Ya*, and 1266 genes and 2098 PAVs from *T. atkins* were added to the reference genome. For the identification of CC-NBS-LRR (CNL) genes in these mango cultivars, this draft pan-genome study has successfully identified 47, 27, and 36 members in *Alphonso*, *H. Xiang Ya*, and *T. atkins* respectively. The phylogenetic analysis divided MiCNL proteins into four distinct subgroups. All *MiCNL* genes are unevenly distributed on chromosomes. Both tandem and segmental duplication events played a significant role in the expansion of the CNL gene family. These genes contain *cis*-elements related to light, stress, hormone, and development. The analysis of protein-protein interactions (PPI) revealed that MiCNL proteins interacted with other defense-responsive proteins. Gene Ontology (GO) analysis indicated that MiCNL genes play a role in defense mechanisms within the organism. The expression level of the identified genes in fruit peel was observed under disease and cold stress which showed that *Mi_A_CNL13* and *14* were up-regulated while *Mi_A_CNL15, 25, 30, 31*, and *40* were down-regulated in disease stress. On the other hand, *Mi_A_CNL2, 14, 41*, and *45* were up-regulated and *Mi_A_CNL47* is down-regulated in cold stress. Subsequently, the Random Forest (RF) classifier was used to assess the multi-stress response of *MiCNLs*. It was found that *Mi_A_CNL14* is a gene that responds to multiple stress conditions. The CNLs have similar protein structures which show that they are involved in the same function. The above findings provide a foundation for a deeper understanding of the functional characteristics of the mango CNL gene family.

## 1 Introduction

Plants have evolved various mechanisms to protect themselves from both biotic and abiotic stresses (Haak et al., 2017). When they are attacked by pathogens, such as bacteria, viruses, fungi, nematodes, and insects, plants activate their pathogen response mechanisms to prevent further harm (Baker et al., 2010). One key component of this defense system is the plant disease resistance (R) genes. These genes play a role in defense against pathogens and are triggered by pathogen signaling (Belkhadir et al., 2004). They can target specific pathogens and are typically encoded by a type of protein called a nucleotide-binding site-leucine-rich repeat (NBS-LRR) protein. The NBS domain of this protein contains three key motifs: the P-loop, kinase-2, and kinase-3a-binding nucleotide (Tameling et al., 2002). The LRR domain, which typically contains 20-30 amino acid residues, is made up of two segments: a highly conserved segment (HCS) and a variable segment (VS) (Matsushima and Miyashita, 2012). The NBS-LRR gene family is the largest class of R genes and plays multiple roles in host-pathogen recognition and downstream signaling transduction (Wan et al., 2012).

NBS-LRR proteins are a class of plant resistance (R) genes that play a crucial role in protecting plants against pathogens. These proteins are divided into two types based on their conserved functional domains: TIR-domain-containing (TNL) and non-TIR-domain-containing. The non-TIR-domain-containing type, also known as CC-NBS-LRR (CNLs), is characterized by the presence of a coiled-coil domain at the N-terminal instead of a TIR domain (Sukarta et al., 2016). Additionally, other domains such as zinc fingers or RPW8 domains may also be present in the N-terminal of CNL genes. CNL genes are found in both monocotyledons and dicotyledons and are widely present in plants (Tarr et al., 2009).

Furthermore, a large proportion of R genes (approx. 80%) encode the NBS-LRR domain, and more than 50 NBS genes have been shown to play a role in disease resistance (Song et al., 2015). Examples of NBS-LRR proteins include the Pi-ta gene in rice, which directly interacts with the Magnaporthe grisea effector AVR-Pita, and the RRS1 protein in *Arabidopsis thaliana*, which directly interacts with the bacterial wilt pathogen protein PopP2 (Jia et al., 2000; Deslandes et al., 2003). Additionally, RPS2 and RPM1 resistance genes in Arabidopsis respond to *Pseudomonas syringae* through indirect interaction with AvrRpm1 and AvrB (DeYoung and Innes, 2006; Gururani et al., 2012). Furthermore, the ectopic overexpression of the Arabidopsis RPW8 gene has been shown to enhance resistance to powdery mildew in grapevine (Hu et al., 2018).

*Mangifera indica* (Mango) belongs to the Anacardiaceae family, which comprises 73 genera and almost 850 species. This fruit grows in tropical and subtropical regions of the world. Mangoes are renowned for being a natural source of dietary fiber, vitamins, proteins, carbohydrates, and essential minerals. They also have a unique flavor and are very nutritious. Therefore, it is called as “King of Tropical Fruits”. Green, yellow, dark red, and orange are the skin colors of ripe mango fruits (Quintana et al., 2021). The mango’s genome was sequenced in 2020, opening up greater resources for molecular studies on this fruit (Wang et al., 2020). The pan-genome of a species encompasses a collection of genes that can be divided into three categories: core genes that are found in all members of the species, accessory genes that are present in some members but not all, and unique genes that are specific to certain individuals within the species. This concept refers to the genetic diversity within a species, rather than an individual genome.

Since CNLs are involved in the defense mechanism of plants against various pathogens including viruses, bacteria, and fungi, the identification of mango CNLs is necessary to understand their interaction mechanisms and to develop defense-resilient cultivars. Additionally, mangoes are traded internationally, and the presence of diseases can restrict exports due to phytosanitary regulations. Disease resistant mango varieties can open up new markets and enhance international trade opportunities.

In this study, only those mango cultivars were chosen that have both the genome and annotation files available. Using a draft pan-genome, the CNL gene family members were identified in three mango cultivars: *Alphonso*, *Hong Xiang Ya*, and *Tommy atkins.* The structural and functional characteristics, gene structure and motifs, chromosomal distribution, gene duplication, *c*is-regulatory elements, protein-protein interaction (PPI), and the expression pattern of *Mi_A_CNLs* at various conditions were analyzed. Furthermore, machine learning techniques were used to identify the multi-stress responsive genes. These results provide worthy clues for further analyzing the biological functions of *MiCNLs* in various other biotic and abiotic stresses.

## 2 Materials and methods

### 2.1 Construction of mango draft pan-genome

The published genomes of three *Mangifera indica* cultivars named *Alphonso, H. Xiang Ya*, and *T. atkins* were downloaded from the MangoBase database (https://mangobase.org/easy_gdb/index.php) (Gómez-Ollé et al., 2023) and a draft pan-genome was constructed based on presence-absence variations (PAVs) using ppsPCP: a plant presence/absence variants scanner and pan-genome construction pipeline (http://cbi.hzau.edu.cn/ppsPCP/) (Ul Qamar et al., 2019). PAVs are the types of Structural Variations (SVs) that are either present or absent in different organisms/genomes. Usually, plants have a PAV length of 100bp. The query genomes were iteratively mapped against reference genome using MUMmer and PAVs were harvested. Next, the harvested PAVs were validated with BLASTn search between the query and reference genomes. Finally, the boundaries of filtered PAVs were corrected and a draft pan-genome was established.

### 2.2 Identification and physiochemical characterization of mango CNLs

The 51 A*. thaliana* CNL protein sequences were retrieved from the Ensembl Plants database (https://plants.ensembl.org/index.html) and a tBLASTn search was performed against the draft pan-genome. From the coordinates of each blast hit, using a draft pan-genome GFF file the protein IDs were obtained and protein sequences were retrieved from the proteome of each cultivar. The identified proteins were further searched for the confirmation of the presence of the NB-ARC and LRR domains in Pfam (http://pfam-legacy.xfam.org/) (Bateman et al., 2004), InterPro (https://www.ebi.ac.uk/interpro/) (Hunter et al., 2009), Conserved Domains Database (CDD; https://www.ncbi.nlm.nih.gov/Structure/cdd/cdd.shtml) (Marchler-Bauer et al., 2015), and HMMER (https://www.ebi.ac.uk/Tools/hmmer/) (Finn et al., 2011) databases. In addition, the coiled-coils structure was confirmed on the Paircoil2 website (https://cb.csail.mit.edu/cb/paircoil2/paircoil2.html), and the P-value parameter was set as 0.025 (McDonnell et al., 2006). The proteins having no characteristic conserved domains were excluded from further analysis.

Physicochemical properties including the length of protein sequence (aa), molecular weight (MW), isoelectric point (pI), aliphatic index (AI), Instability index (II), and grand average of hydropathicity (GRAVY) values were predicted using ProtParam tool of Expasy server (https://web.expasy.org/protparam/) (Gasteiger et al., 2005). Additionally, subcellular localization of mango CNL proteins was predicted using an online WoLF PSORT tool (https://wolfpsort.hgc.jp/) (Horton et al., 2007).

### 2.3 Multiple sequence alignment, phylogenetic analysis, conserved motifs, and gene structure analysis of MiCNLs

To further evaluate the evolutionary link of CNL proteins, a multiple sequence alignment of 51 A*. thaliana* (AtCNLs), 33 *Cucumis sativus L.* (CsaCNLs), 10 *Citrus sinensis* (ScCNLs), 47 *Alphonso* (Mi_A_CNLs), 27 *H. Xiang Ya* (Mi_H_CNLs), and 36 *T. atkins* (Mi_T_CNLs) protein were completed using ClustalW program (Tamura et al., 2021), and a phylogenetic tree was constructed using IQTREE Web Server (http://iqtree.cibiv.univie.ac.at/) (Zameer et al., 2021). The reliability of the constructed tree was verified using 1000 bootstrapping replicates using the maximum likelihood (ML) method. The tree was further edited using the iTOL: Interactive Tree of Life (https://itol.embl.de/) (Letunic and Bork, 2021).

To find common motifs among each mango cultivar, the Multiple Expectation Maximization for Motif Elicitation tool (MEME, https://meme-suite.org/meme/) (Bailey et al., 2015) was applied using protein sequences. Except for setting the motif number to 20, the rest of the parameters were retained by default. TBtools was used to visualize the identified motifs. The GFF file of each mango cultivar was used to analyze the intron and exon pattern of *MiCNL* genes and the structures were displayed using TBtools (Chen et al., 2018).

### 2.4 Chromosomal localization, Ka/Ks, and gene duplication analysis

The chromosomal position of each *MiCNL* gene was acquired from the GFF file of the relative cultivar and mapped using the gene location visualization tool of TBtools software (Chen et al., 2018). *MiCNL* gene duplication events were determined based on whether the length of the shorter gene covered was equal to or greater than 70% of the longer gene and if the similarity of the two aligned genes was equal to or greater than 70% (Tsai et al., 2012). Tandem and segmental duplications are reported to be the two main mechanisms underlying gene family expansion. Genes located on the same chromosome fragment were considered to be tandem duplicated genes. Genes found to be co-paralogs located on duplicated chromosomal blocks were considered to be segmentally duplicated genes (Flagel and Wendel, 2009). Ka/Ks values can be used to predict selection pressure for replicating genes. DnaSP v.6 software (Rozas et al., 2017) was used to calculate the nonsynonymous (Ka) and synonymous (Ks) nucleotide substitution parameters. If the ratio of Ka/Ks was greater than, equal to, or less than one, this indicated positive, neutral, and purifying selection, respectively (Zia et al., 2022). Moreover, the time of divergence for these gene pairs was calculated using the formula “t = Ks/2λ×10^-6^”, with λ value of 1.5× 10^−8^ for dicots to calculate the duplication time in million years (Zameer et al., 2022; Sadaqat et al., 2023).

### 2.5 *Cis*-regulatory elements, protein-protein interaction, and gene ontology enrichment analysis

As in the earlier studies, the *cis*-acting elements in the 2,000 bp upstream sequences in the genomic region of *MiCNL* genes were retrieved from the genome file using the “samtools faidx” tool in Ubuntu (Li et al., 2009; Hu et al., 2022; Xia et al., 2022; Zhu et al., 2022), and the types, numbers, and functions of these elements were analyzed using PlantCARE database (https://bioinformatics.psb.ugent.be/webtools/plantcare/html/) (Rombauts et al., 1999). *Cis*-elements were visualized using TBtools software.

Protein sequences of MiCNL were used as input in the STRING database (https://string-db.org/) (Mering et al., 2003) for analyzing PPI. For PPI the level of connection used was tenth and other parameters were kept by default. The PPI network was visualized and edited using Cytoscape software (Shannon et al., 2003). GO enrichment analysis was done using the DAVID database (https://david.ncifcrf.gov/home.jsp) (Dennis et al., 2003) and the components considered were biological processes (BP), cellular components (CC), and molecular function, and KEGG pathways.

### 2.6 Tissue specific analysis and 3D structure prediction of Mi_A_CNLs

The expression levels of all *Mi_A_CNL* genes under disease and cold stress were evaluated using transcriptome datasets available at the NCBI Sequence Read Archive (SRA) database (https://www.ncbi.nlm.nih.gov/sra) (Kodama et al., 2012) under BioProject: PRJNA855362 and PRJNA304093 respectively. The genome and annotation files (GFF) were downloaded from the MangoBase database (https://mangobase.org/easy_gdb/index.php) (Gómez-Ollé et al., 2023). The reads quality was checked through the FastQC tool (Brown et al., 2017). Indexes of *M. indica* (*Alphonso*) genome sequences were built using Bowtie2 (Langdon, 2015) and high-quality paired-end reads were mapped to the genome. The Htseq-count (Anders et al., 2015) program used abundance estimation of annotated genes. Finally, count values of individual genes were used to generate the heatmap which was illustrated using TBtools software.

To function properly, proteins are needed to be folded into a proper three-dimensional structure. Based on expression patterns, four *Mi_A_CNL* proteins were selected to predict their structures. Alphafold2 (https://rb.gy/dlamz) was used for this purpose (Jumper et al., 2021). Further, the predicted structures were validated using SAVES (https://saves.mbi.ucla.edu/) (Sawal et al., 2023) and MolProbity (http://molprobity.biochem.duke.edu/) (Davis et al., 2007). PyMOL was used to visualize these structures (Alexander et al., 2011).

### 2.7 Prediction of multi-stress responsive genes using machine learning

DESeq2 was utilized to investigate both disease and cold stress samples to identify genes with significant expression changes (Anders and Huber, 2012). Based on statistical significance, the identified genes were screened based on their p-value < 0.05 and log2 fold change values (a log2FC value ≥ 0.5 for upregulation, and log2FC ≤ -0.5 for downregulation). Common CNL genes from both datasets were used for testing. To verify the validity of these genes, the random forest (RF) classification algorithm was applied within the R programming environment (Qi, 2012). Model performance assessment usually involves a comparison of the model’s predictions with the known values of the dependent variable within a specific dataset. Count values of disease datasets were taken to train the model and common genes were used for testing. Performance metrics such as accuracy, area under the receiver operating characteristic curve (AUC), specificity, and sensitivity were used to evaluate the effectiveness of the RF classifier, specifically on the dataset containing common multi-stress responsive gene.

## 3 Results

### 3.1 Draft pan-genome of three mango cultivars

**Figure 1:**
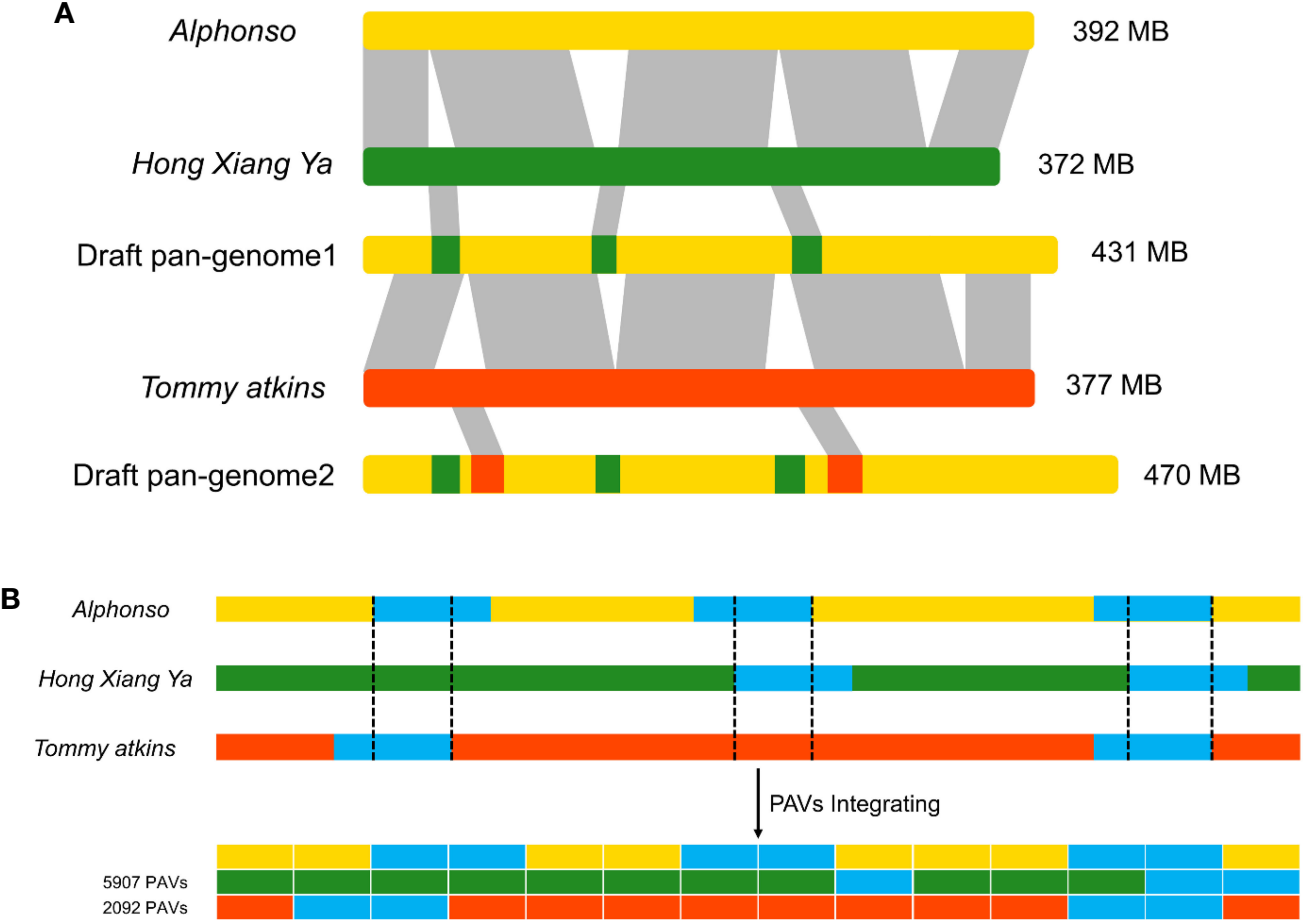
Construction of linearized draft pan-genome. **(A)** Genomes used for draft pan-genome construction are iteratively aligned between genomes and the starting reference to identify novel segments, then integrate these sequences into the reference genome to construct a draft pan-genome. **(B)** PAVs scanning and genotyping in the draft pan-genome.

Three mango genomes of cultivars: *Alphonso*, *H. Xiang Ya*, and *T. atkins* were used to construct a draft pan-genome through ppsPCP. The *Alphonso* genome was selected as a reference based on its quality and completeness, while *H. Xiang Ya* and *T. atkins* were mapped iteratively against the selected reference genome. In the first iteration, the *H. Xiang Ya* genome contributed 5907 PAVs and 2823 new genes to the reference genome. While, in the second iteration, *T. atkins* contributed 2092 PAVs and 1266 new genes to the developing draft pan-genome (**Table S1**). In total, 7999 novel PAVs and 4089 new genes were added to the reference genome and a draft pan-genome assembly was established (**Figure 1**). The total draft pan-genome assembly size was 470 MB, with a total of 39843 genes in its annotation file. The draft pan-genome assembly fasta (.fa) and annotation (.gff3) files are given in **Supplementary Material**.

### 3.2 Identification and physiochemical characteristics of CNL genes in *Mangifera indica* cultivars

**Figure 2:**
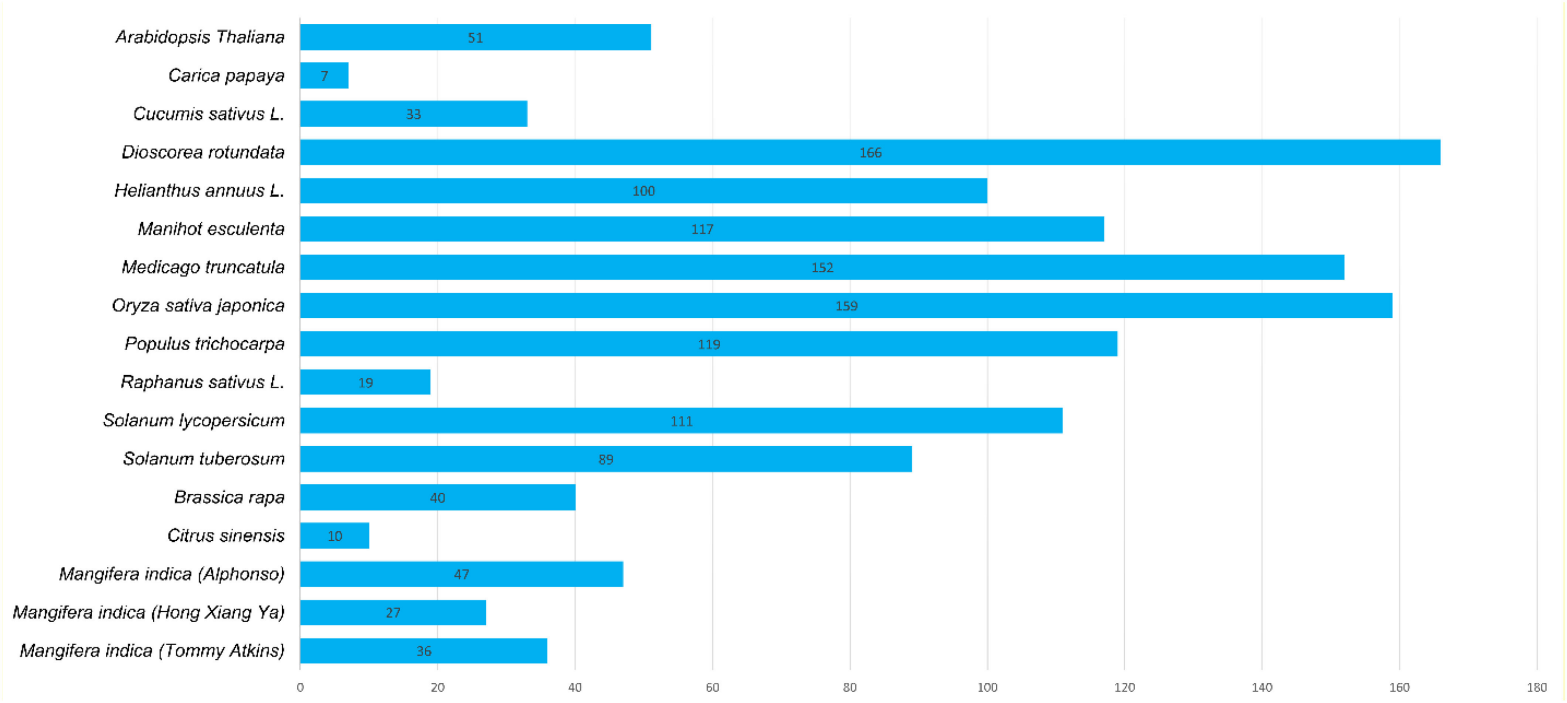
Identified CNL gene family members from *Mangifera indica* cultivars and other plant species.

A total of 47, 27, and 36 CNL genes were identified from the genomes of *Alphonso* (*Mi_A_CNLs*), *H. Xiang Ya* (*Mi_H_CNLs*), and *T. atkins* (*Mi_T_CNLs*), respectively. All of the identified MiCNLs were also confirmed for the presence of coil-coil, NB-ARC, and LRR domains (**Table S2**). The CNLs in *Mangifera indica* cultivars were relatively less than *A. thaliana*, *Oryza sativa*, *Medicago truncatula*, *Helianthus annuus L.*, and *Dioscorea rotundata* but higher than *C. sinensis*, *Brassica rapa*, *Cucumis sativus*, and *Raphanus sativus* (**Figure 2**).

The protein names of each cultivar were named from CNL1 onward according to their position on chromosomes, from Chr1 to Chr20 (**Table 1**).

**Table 1:** Details of identified *CNLs* in *M. indica* cultivars.

Gene Name	Gene ID	Group	Chromosome	Start	End	Strand	Subcellular localization
*M. indica (Alphonso)*							
*Mi_A_CNL1*	LOC123220599	A	Chr1	5711589	5715357	–	Cytoplasm
*Mi_A_CNL2*	LOC123209381	A	Chr1	5730642	5734541	–	Nucleus
*Mi_A_CNL3*	LOC123218214	C	Chr1	9196496	9199407	+	Cytoplasm
*Mi_A_CNL4*	LOC123215178	C	Chr1	9313244	9316694	+	Chloroplast
*Mi_A_CNL5*	LOC123219339	B	Chr1	27270128	27273740	+	Nucleus
*Mi_A_CNL6*	LOC123213767	C	Chr1	28915672	28920915	–	Nucleus
*Mi_A_CNL7*	LOC123209617	C	Chr2	14187952	14192623	+	Nucleus
*Mi_A_CNL8*	LOC123204063	C	Chr2	14368299	14371227	–	Nucleus
*Mi_A_CNL9*	LOC123208576	C	Chr2	14375878	14378458	–	Chloroplast
*Mi_A_CNL10*	LOC123204072	C	Chr2	14382559	14385212	+	Chloroplast
*Mi_A_CNL11*	LOC123208577	C	Chr2	14387150	14389735	+	Chloroplast
*Mi_A_CNL12*	LOC123208579	C	Chr2	14410183	14414603	+	Cytoplasm
*Mi_A_CNL13*	LOC123201503	B	Chr2	22594224	22599836	–	Chloroplast
*Mi_A_CNL14*	LOC123209058	B	Chr2	22602336	22607922	–	Nucleus
*Mi_A_CNL15*	LOC123210654	B	Chr3	6069550	6077011	–	Cytoplasm
*Mi_A_CNL16*	LOC123210656	B	Chr3	6088460	6091345	–	Cytoplasm
*Mi_A_CNL17*	LOC123211055	B	Chr3	6646374	6650817	–	Chloroplast
*Mi_A_CNL18*	LOC123211057	B	Chr3	6653858	6657623	–	Nucleus
*Mi_A_CNL19*	LOC123211058	B	Chr3	6661007	6664607	–	Nucleus
*Mi_A_CNL20*	LOC123211060	B	Chr3	6670524	6674217	–	Nucleus
*Mi_A_CNL21*	LOC123211212	B	Chr3	7021234	7024534	+	Chloroplast
*Mi_A_CNL22*	LOC123211877	B	Chr3	18502532	18518194	–	Cytoplasm
*Mi_A_CNL23*	LOC123211635	B	Chr3	21749856	21752705	+	Nucleus
*Mi_A_CNL24*	LOC123214379	C	Chr4	20658197	20661620	–	Cytoplasm
*Mi_A_CNL25*	LOC123214535	C	Chr4	20890875	20896330	+	Nucleus
*Mi_A_CNL26*	LOC123216372	C	Chr5	12883506	12887432	+	Nucleus
*Mi_A_CNL27*	LOC123219383	C	Chr6	7018300	7021626	+	Cytoplasm
*Mi_A_CNL28*	LOC123221139	C	Chr7	2885384	2889217	–	Cytoplasm
*Mi_A_CNL29*	LOC123228245	A	Chr10	398100	401366	+	Chloroplast
*Mi_A_CNL30*	LOC123229776	A	Chr11	2592597	2596313	–	Nucleus
*Mi_A_CNL31*	LOC123229777	A	Chr11	2599730	2603343	–	Nucleus
*Mi_A_CNL32*	LOC123192330	C	Chr12	6766637	6772521	–	Nucleus
*Mi_A_CNL33*	LOC123196029	C	Chr14	2450442	2453601	–	Endoplasmic reticulum
*Mi_A_CNL34*	LOC123195951	C	Chr14	2472407	2475919	+	Chloroplast
*Mi_A_CNL35*	LOC123199462	C	Chr16	1321923	1325404	–	Nucleus
*Mi_A_CNL36*	LOC123199183	A	Chr16	2262652	2271819	+	Nucleus
*Mi_A_CNL37*	LOC123199184	A	Chr16	2279091	2283292	+	Nucleus
*Mi_A_CNL38*	LOC123198840	C	Chr16	13127703	13134495	+	Nucleus
*Mi_A_CNL39*	LOC123199400	C	Chr16	13146385	13150951	+	Nucleus
*Mi_A_CNL40*	LOC123199894	C	Chr17	3683551	3709400	–	Cytoplasm
*Mi_A_CNL41*	LOC123199895	C	Chr17	3729336	3757669	–	Cytoplasm
*Mi_A_CNL42*	LOC123200365	C	Chr17	10906610	10909877	+	Chloroplast
*Mi_A_CNL43*	LOC123200233	C	Chr17	10958224	10960998	+	Cytoplasm
*Mi_A_CNL44*	LOC123199961	C	Chr17	10985385	10988251	+	Cytoplasm
*Mi_A_CNL45*	LOC123202131	B	Chr18	12982214	12985341	–	Nucleus
*Mi_A_CNL46*	LOC123203903	C	Chr20	10954280	10957614	–	Chloroplast
*Mi_A_CNL47*	LOC123203859	C	Chr20	10996927	11000057	–	Endoplasmic reticulum
*M. indica (Hong Xiang Ya)*							
*Mi_H_CNL1*	GWHGABLA018645	C	Chr2	2181546	2184782	–	Cytoplasm
*Mi_H_CNL2*	GWHGABLA018663	B	Chr2	2488114	2492313	+	Chloroplast
*Mi_H_CNL3*	GWHGABLA018787	D	Chr2	4128718	4131587	+	Chloroplast
*Mi_H_CNL4*	GWHGABLA024667	B	Chr4	6555478	6561794	–	Nucleus
*Mi_H_CNL5*	GWHGABLA024671	B	Chr4	6575208	6577890	–	Nucleus
*Mi_H_CNL6*	GWHGABLA024672	B	Chr4	6581773	6585107	–	Nucleus
*Mi_H_CNL7*	GWHGABLA027774	C	Chr6	1255905	1258921	–	Cytoplasm
*Mi_H_CNL8*	GWHGABLA027891	A	Chr6	2110435	2117173	+	Nucleus
*Mi_H_CNL9*	GWHGABLA027892	A	Chr6	2124012	2143734	+	Nucleus
*Mi_H_CNL10*	GWHGABLA029891	C	Chr7	14631040	14634411	+	Endoplasmic reticulum
*Mi_H_CNL11*	GWHGABLA002040	C	Chr10	8324106	8326913	–	Endoplasmic reticulum
*Mi_H_CNL12*	GWHGABLA002331	D	Chr10	11173251	11175860	–	Nucleus
*Mi_H_CNL13*	GWHGABLA002335	D	Chr10	11202852	11205713	–	Cytoplasm
*Mi_H_CNL14*	GWHGABLA002469	B	Chr10	12214032	12216701	–	Cytoplasm
*Mi_H_CNL15*	GWHGABLA002470	B	Chr10	12231470	12234153	–	Nucleus
*Mi_H_CNL16*	GWHGABLA005206	C	Chr12	2527497	2530340	–	Cytoplasm
*Mi_H_CNL17*	GWHGABLA005208	C	Chr12	2550148	2552999	–	Endoplasmic reticulum
*Mi_H_CNL18*	GWHGABLA006200	B	Chr12	13042723	13048093	+	Nucleus
*Mi_H_CNL19*	GWHGABLA006205	B	Chr12	13156112	13161109	+	Nucleus
*Mi_H_CNL20*	GWHGABLA006208	B	Chr12	13224145	13229537	+	Nucleus
*Mi_H_CNL21*	GWHGABLA009646	A	Chr15	2604702	2606388	–	Cytoplasm
*Mi_H_CNL22*	GWHGABLA009647	A	Chr15	2611124	2613816	–	Nucleus
*Mi_H_CNL23*	GWHGABLA011611	C	Chr16	11915477	11918558	+	Chloroplast
*Mi_H_CNL24*	GWHGABLA015785	C	Chr18	21418990	21421374	–	Chloroplast
*Mi_H_CNL25*	GWHGABLA016656	A	Chr19	5433399	5436916	–	Nucleus
*Mi_H_CNL26*	GWHGABLA018144	B	Chr19	25721920	25724934	+	Chloroplast
*Mi_H_CNL27*	GWHGABLA018380	C	Chr19	27532069	27534275	–	Nucleus
*M. indica (Tommy Atkins)*							
*Mi_T_CNL1*	Manin02g000840	C	Chr2	1229173	1232869	–	Endoplasmic reticulum
*Mi_T_CNL2*	Manin03g005110	C	Chr3	9896519	9899590	–	Nucleus
*Mi_T_CNL3*	Manin03g005120	C	Chr3	9904392	9906972	–	Chloroplast
*Mi_T_CNL4*	Manin03g005130	C	Chr3	9911591	9919202	+	Endoplasmic reticulum
*Mi_T_CNL5*	Manin03g005150	C	Chr3	9939617	9944041	+	Cytoplasm
*Mi_T_CNL6*	Manin04g007540	B	Chr4	5754414	5759283	–	Cytoplasm
*Mi_T_CNL7*	Manin04g008210	B	Chr4	6256215	6268375	–	Nucleus
*Mi_T_CNL8*	Manin04g016160	B	Chr4	18022955	18025714	–	Cytoplasm
*Mi_T_CNL9*	Manin06g001600	C	Chr6	1253620	1261613	–	Nucleus
*Mi_T_CNL10*	Manin07g007570	C	Chr7	10534921	10537638	+	Cytoplasm
*Mi_T_CNL11*	Manin07g007580	C	Chr7	10560144	10562849	+	Cytoplasm
*Mi_T_CNL12*	Manin07g007800	C	Chr7	10870672	10875128	+	Nucleus
*Mi_T_CNL13*	Manin10g008560	B	Chr10	10775434	10778355	–	Nucleus
*Mi_T_CNL14*	Manin10g008590	B	Chr10	10818174	10820843	–	Nucleus
*Mi_T_CNL15*	Manin10g008600	B	Chr10	10835939	10839756	–	Nucleus
*Mi_T_CNL16*	Manin12g002700	C	Chr12	2230962	2233805	–	Chloroplast
*Mi_T_CNL17*	Manin12g002730	C	Chr12	2253605	2256448	–	Chloroplast
*Mi_T_CNL18*	Manin12g002740	C	Chr12	2265725	2269856	–	Endoplasmic reticulum
*Mi_T_CNL19*	Manin12g002750	C	Chr12	2282982	2292228	+	Nucleus
*Mi_T_CNL20*	Manin13g010790	C	Chr13	12298716	12301259	–	Nucleus
*Mi_T_CNL21*	Manin15g003400	A	Chr15	2638486	2655784	–	Chloroplast
*Mi_T_CNL22*	Manin15g003410	A	Chr15	2655999	2657909	–	Nucleus
*Mi_T_CNL23*	Manin16g007090	C	Chr16	13284305	13299820	+	Nucleus
*Mi_T_CNL24*	Manin17g007260	C	Chr17	10678513	10681296	–	Chloroplast
*Mi_T_CNL25*	Manin18g001880	C	Chr18	1229671	1250876	+	Chloroplast
*Mi_T_CNL26*	Manin18g010170	C	Chr18	9870715	9873417	+	Nucleus
*Mi_T_CNL27*	Manin19g006820	A	Chr19	5429993	5433782	–	Nucleus
*Mi_T_CNL28*	Manin19g009550	C	Chr19	8844254	8846864	+	Cytoplasm
*Mi_T_CNL29*	Manin19g009570	C	Chr19	8891536	8894145	+	Nucleus
*Mi_T_CNL30*	Manin19g009590	C	Chr19	8929056	8936542	+	Nucleus
*Mi_T_CNL31*	Manin19g009600	C	Chr19	8944885	8947491	+	Nucleus
*Mi_T_CNL32*	Manin19g014760	B	Chr19	20321125	20323863	+	Chloroplast
*Mi_T_CNL33*	Manin00g008100	C	10000001	23913927	23917394	–	Cytoplasm
*Mi_T_CNL34*	Manin00g008730	C	10000001	25320996	25326389	–	Nucleus
*Mi_T_CNL35*	Manin00g008930	B	10000001	25583732	25586779	–	Nucleus
*Mi_T_CNL36*	Manin00g017170	C	10000001	45958939	45968686	+	Endoplasmic reticulum

**Figure 3:**
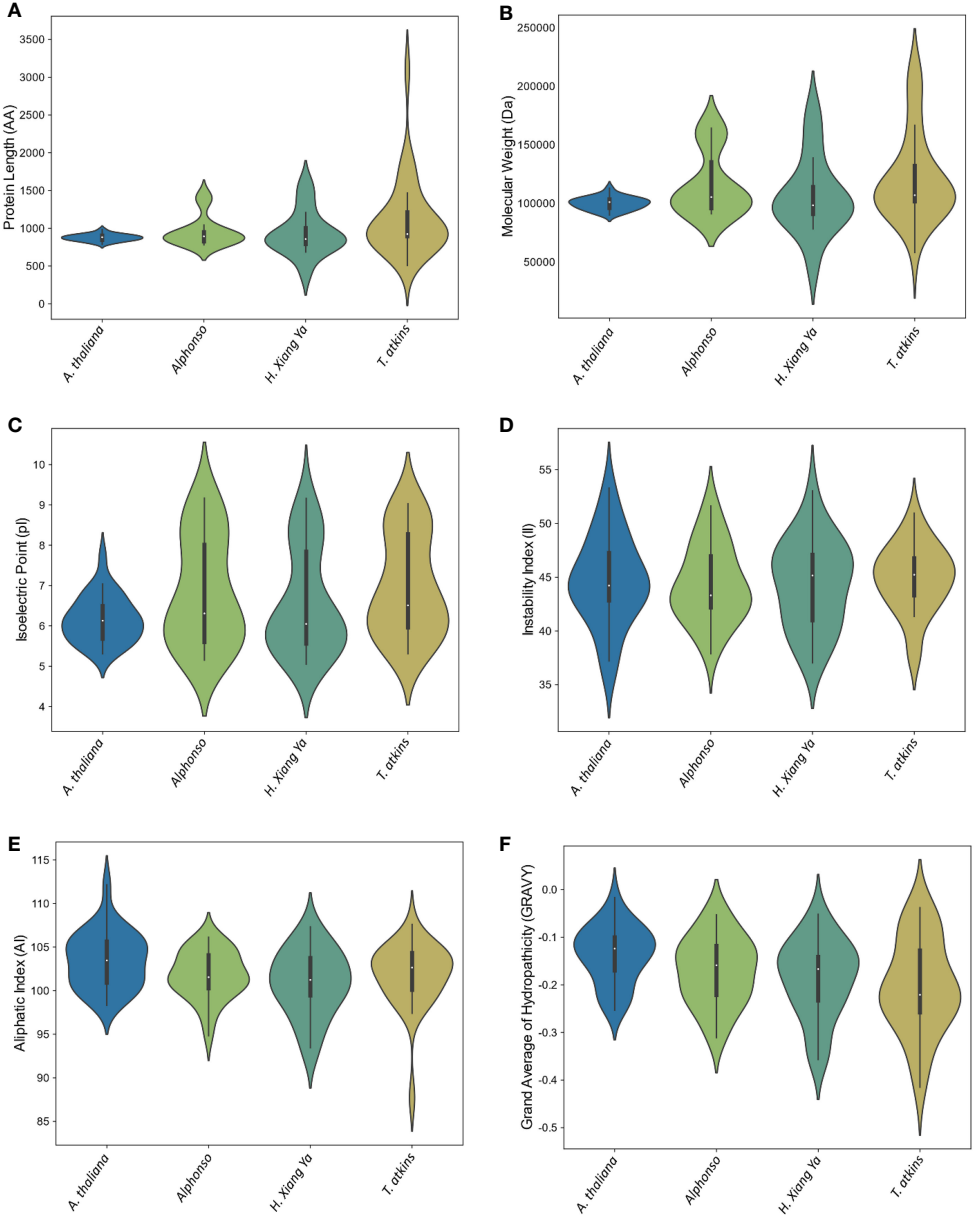
Violin plot of physiochemical properties of *A thaliana* and three *M. indica* cultivars. **(A)** Protein length, **(B)** Molecular weight, **(C)** Isoelectric point, **(D)** Instability index, **(E)** Aliphatic Index, and **(F)** Grand average of hydropathicity (GRAVY).

The physical and chemical properties of all MiCNL proteins were analyzed (**Table S3**). There were no significant differences in amino acid residue number, molecular weights, isoelectric point instability index, aliphatic index, and GRAVY among the three cultivars. In all cultivars, most of the proteins have an isoelectric point (pI) less than 7 indicating that these proteins have acidic behavior. The instability index (II) values of most proteins indicated that these are unstable in the test tube. Most of the proteins have an aliphatic index (AI) greater than 70 which indicates that these proteins are thermally stable, and negative GRAVY values indicate that these proteins are hydrophilic (**Figure 3**). The protein’s subcellular localization shows that most of the proteins were present in the cytoplasm and nucleus. Few proteins were present in the chloroplast and endoplasmic reticulum (**Table 1**).

### 3.3 Phylogenetic relationships of CNL family members from three *M. indica* cultivars

**Figure 4:**
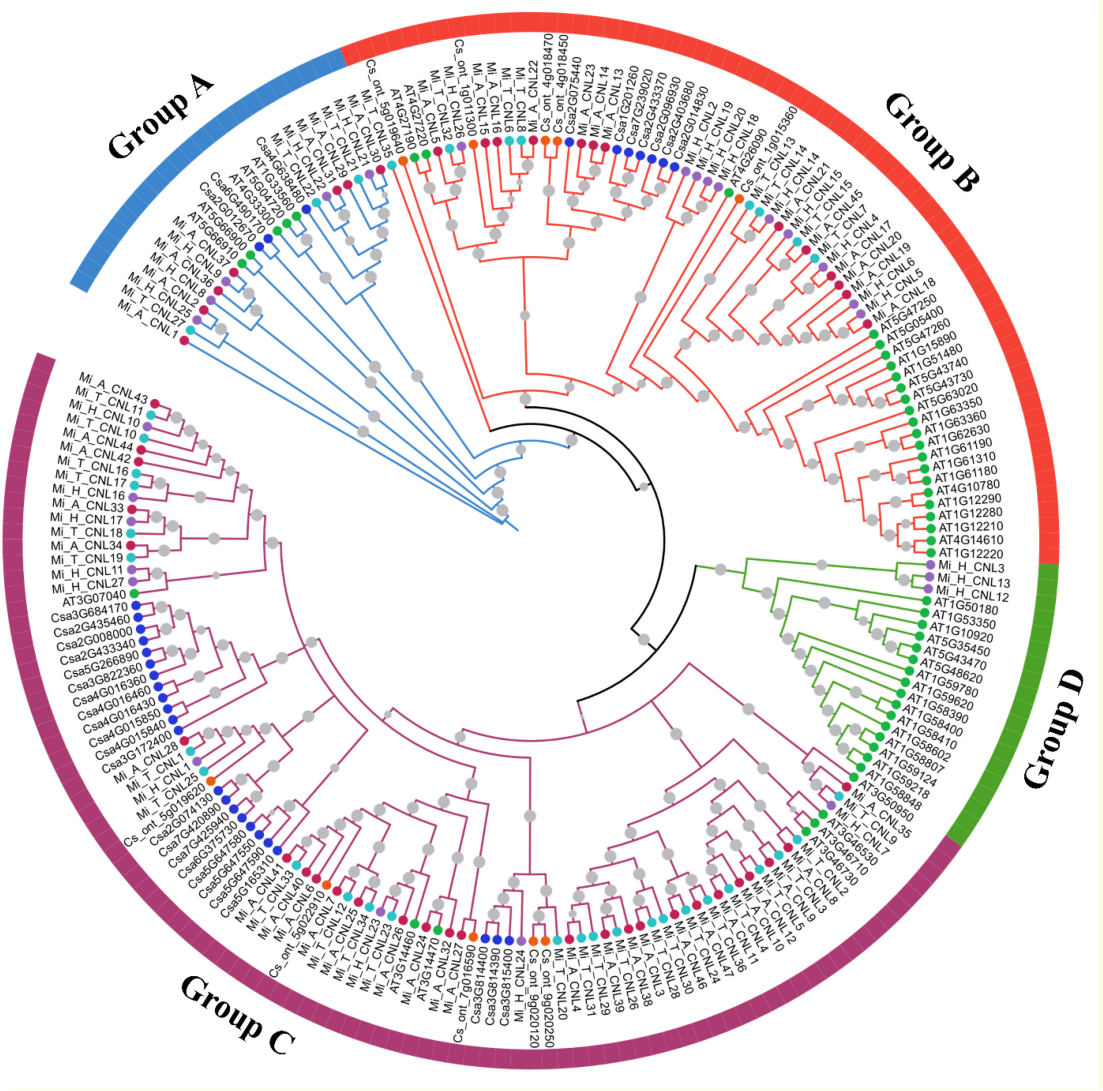
A maximum likelihood (ML) phylogenetic tree of CNL protein sequences from *A. thaliana* (At), *C. sativus* (Csa), *C. sinensis*, *M. indica Alphonso* (Mi_A_CNLs), *M. indica H. Xiang Ya* (Mi_H_CNLs), and *M. indica T. atkins* (Mi_T_CNLs). Different colors of branches represent different groups.

To analyze the possible evolutionary relationship of the CNL gene family in *M. indica* cultivars, a phylogenetic tree was constructed using 204 amino acid sequences from six species. All CNL proteins were clustered into four groups. In comparison, group C contained the most CNL gene family members including 27 Mi_A_CNLs, 9 Mi_H_CNLs, 25 Mi_T_CNLs, 7 AtCNLs, 5 CsCNLs, and 23 CsaCNLs followed by group B which contain 13 Mi_A_CNLs, 10 Mi_H_CNLs, 8 Mi_T_CNLs, 23 AtCNLs, 5 CsCNLs, and 7 CsaCNLs. Group A contains 7 Mi_A_CNLs, 5 Mi_H_CNLs, 3 Mi_T_CNLs, 5 AtCNLs, and 3 CsaCNLs. Group D had only 3 members of Mi_H_CNLs and 16 members of AtCNLs. No member of Mi_A_CNLs, Mi_T_CNLs, CsCNLs and CsaCNLs was present in group D (**Figure 4**).

### 3.4 Conserved motifs, and gene structure analysis of *MiCNLs*

**Figure 5:**
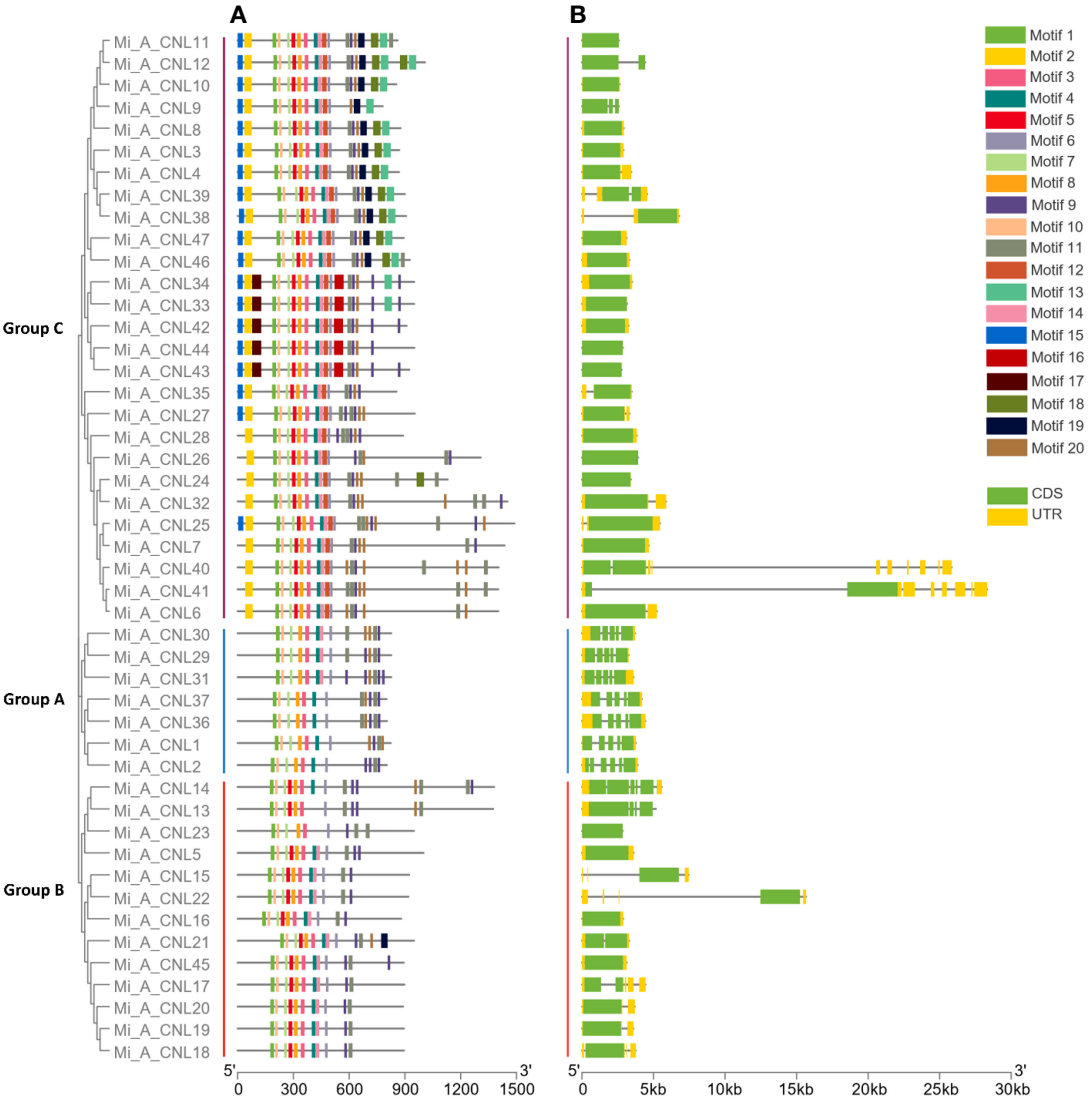
Phylogenetic tree, motif pattern, and gene structure of *Mi_A_CNLs* constructed using TBtools software. **(A)** Conserved motifs were determined using the MEME suite. **(B)** Gene structure was determined to show conservation among genes.

Overall, 20 motifs were chosen to analyze the pattern of conserved motifs among the MiCNLs. These motifs were identified through annotation from the Pfam database. The NBS domain consists of 8 motifs. Specifically, motif 1 was identified as the P-loop (Kinase a), motif 3 as GLPL, motif 4 as RNBS-D, motif 6 as MHD, motif 7 as Kinase-2, motif 8 as RNBS-C, motif 10 as RNBS-A, motif 13 as LRR. Out of 20, a total of 12 motifs (1, 3, 4, 6, 7, 8, 9, 10, 11, 13, 14, and 19) were conserved in all proteins of *Alphonso*. Motifs 2 and 12 were only conserved in the members of group C. Motif 5 was conserved in all proteins except the proteins of group A (**Figure 5A**). In *H. Xiang Ya* 8 motifs (1,2,3,4,5,6,7, and 8) were conserved in all proteins expects 2 proteins (Mi_H_CNL21 and Mi_H_CNL23). Motif 18 was only conserved in group B (**Figure S1A**). In *T. atkins* 6 motifs (1, 2, 3, 8, 9, and 12) were conserved among all members. Motifs 5 and 16 were only conserved in the members of group C (**Figure S2A**).

In *Alphonso*, gene structure varies from one group to another group. In group A, all the members have 5 exons and 4 introns. Group B has 1-4 exons and 0-3 introns, while members of Group C have 1-2 exons and 0-1 introns. Most of the members in group C have only 1 exon and no intron (**Figure 5B**). In *H. Xiang Ya* group A exons ranged from 3-13 and introns ranged from 2-12. Group B has 2-5 exons and 1-4 introns. Group C has 1-4 exons and 0-3 introns, while all members of Group D have only 2 exons and 1 intron (**Figure S1B**). In group A, *T. atkins* exons had a range from 5-13 and introns had a range from 4-12. Most of the members of group B had 1 exon but few members had a range of 1-3 exons and 0-2 introns. Members of group C have 1-15 exons and 0-14 introns (**Figure S2B**).

### 3.5 Chromosomal mapping and gene duplication analysis

**Figure 6:**
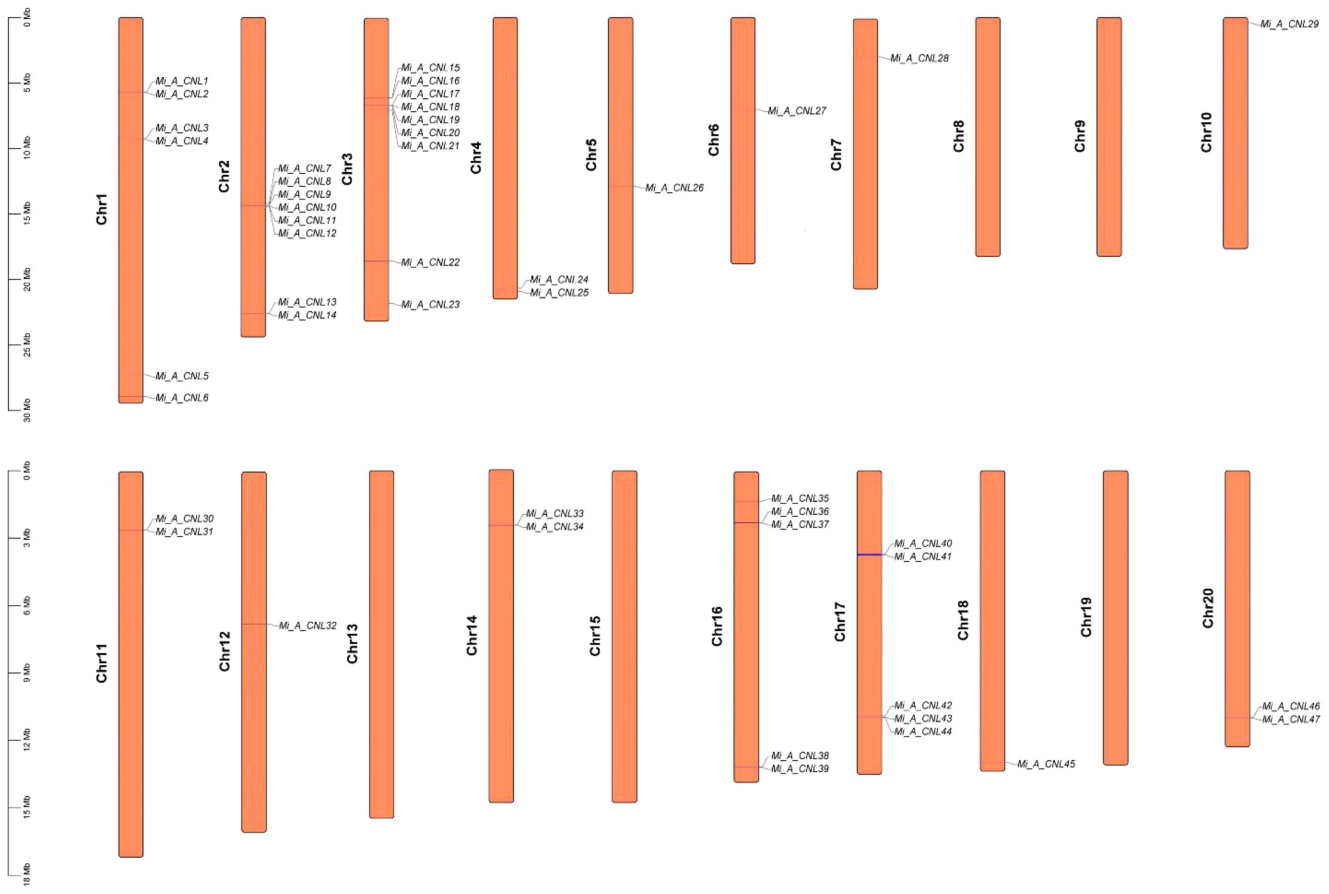
Chromosomal mapping of *Mi_ A_CNL* genes. 15 out of 20 chromosomes contain CNL genes.

In *Alphonso*, 47 CNL genes were distributed unevenly on 15 out of 20 chromosomes. It had maximum genes (9) at Chr3, and minimum genes (1) at Chr5, 6, 7, 10, 12, and 18. There was no gene on Chr 8, 9, 13, 15, and 19 (**Figure 6**). In *H. Xiang Ya* 27 genes were mapped unevenly at 10 out of 20 chromosomes. In this maximum gene (5) were present at chromosome 10 and 12, and minimum gene (1) was present at chromosome 7, 16, and 18. No gene was present at chromosomes 1, 3, 5, 8, 9, 11, 13, 14, and 17 (**Figure S3**). In *T. Atkins* 36 genes were present on 13 out of 20 chromosomes and on the scaffold. Chr19 has the maximum number of genes (5 genes) and Chr2, 6, 13, 16, and 17 have minimum numbers of genes (1 gene) on each chromosome. Four genes *Mi_T_CNL33-36* were present on the scaffold (10000001). No gene was present on Chr1, 5, 8, 9, 11, and 14 (**Figure S4**).

Gene duplication events were also analyzed among *Mi_A_CNL, Mi_H_CNL*, and *Mi_T_CNL* genes and a total of 37, 8, and 5 duplicated pairs of genes were found among all the members respectively. Most of the members were tandemly duplicated. On the other hand, a few members resulted from segmental duplication. Thus, in line with previous studies, these findings indicated that tandem, as well as segmental duplications, were the main factor causing the increase of the CNL gene family in *M. indica* cultivars (**Table 2**).

**Table 2:** Duplication data of three *M. indica* cultivars genes, rate of synonymous and non-synonymous mutations, duplication time (MYA), and type of duplication between genes.

Gene 1	Gene 2	Ka	Ks	Ka/Ks	Time (MYA)	Duplication Type
*Mi_A_CNL1*	*Mi_A_CNL2*	0.0066	0.0103	0.640776699	0.343333333	Tandem
*Mi_A_CNL1*	*Mi_A_CNL35*	2.1205	2.6524	0.799464636	88.41333333	Segmental
*Mi_A_CNL1*	*Mi_A_CNL36*	1.5561	1.6141	0.964066663	53.80333333	Segmental
*Mi_A_CNL2*	*Mi_A_CNL36*	1.5326	1.6705	0.917449865	55.68333333	Segmental
*Mi_A_CNL2*	*Mi_A_CNL37*	1.4916	1.7782	0.838825779	59.27333333	Segmental
*Mi_A_CNL3*	*Mi_A_CNL4*	0.3461	0.4532	0.763680494	15.10666667	Tandem
*Mi_A_CNL3*	*Mi_A_CNL38*	0.3894	0.5596	0.695854182	18.65333333	Segmental
*Mi_A_CNL6*	*Mi_A_CNL40*	0.1019	0.1485	0.686195286	4.95	Segmental
*Mi_A_CNL6*	*Mi_A_CNL41*	0.0938	0.1328	0.706325301	4.426666667	Segmental
*Mi_A_CNL8*	*Mi_A_CNL9*	0.3142	0.4931	0.637193267	16.43666667	Tandem
*Mi_A_CNL8*	*Mi_A_CNL47*	0.4625	0.5737	0.806170472	19.12333333	Segmental
*Mi_A_CNL9*	*Mi_A_CNL10*	2.5202	2.679	0.940724151	89.3	Tandem
*Mi_A_CNL9*	*Mi_A_CNL11*	2.4272	3.0348	0.799789113	101.16	Tandem
*Mi_A_CNL10*	*Mi_A_CNL11*	0.0627	0.1043	0.601150527	3.476666667	Tandem
*Mi_A_CNL10*	*Mi_A_CNL12*	0.0849	0.0789	1.076045627	2.63	Tandem
*Mi_A_CNL11*	*Mi_A_CNL12*	0.0683	0.0933	0.73204716	3.11	Tandem
*Mi_A_CNL13*	*Mi_A_CNL14*	0.0555	0.0796	0.697236181	2.653333333	Tandem
*Mi_A_CNL15*	*Mi_A_CNL16*	0.1156	0.144	0.802777778	4.8	Tandem
*Mi_A_CNL15*	*Mi_A_CNL22*	0.1346	0.1219	1.104183757	4.063333333	Tandem
*Mi_A_CNL16*	*Mi_A_CNL22*	0.1888	0.1577	1.197209892	5.256666667	Tandem
*Mi_A_CNL17*	*Mi_A_CNL18*	0.0778	0.1294	0.601236476	4.313333333	Tandem
*Mi_A_CNL17*	*Mi_A_CNL19*	0.08	0.1323	0.604686319	4.41	Tandem
*Mi_A_CNL17*	*Mi_A_CNL20*	0.0949	0.1555	0.610289389	5.183333333	Tandem
*Mi_A_CNL18*	*Mi_A_CNL19*	0.035	0.0681	0.513950073	2.27	Tandem
*Mi_A_CNL18*	*Mi_A_CNL20*	0.0693	0.1288	0.538043478	4.293333333	Tandem
*Mi_A_CNL19*	*Mi_A_CNL20*	0.0592	0.1112	0.532374101	3.706666667	Tandem
*Mi_A_CNL30*	*Mi_A_CNL31*	0.0785	0.1455	0.5395189	4.85	Tandem
*Mi_A_CNL34*	*Mi_A_CNL42*	0.1428	0.1814	0.787210584	6.046666667	Segmental
*Mi_A_CNL34*	*Mi_A_CNL43*	0.1368	0.2135	0.640749415	7.116666667	Segmental
*Mi_A_CNL34*	*Mi_A_CNL44*	0.1332	0.1885	0.7066313	6.283333333	Segmental
*Mi_A_CNL36*	*Mi_A_CNL37*	0.0298	0.0548	0.54379562	1.826666667	Tandem
*Mi_A_CNL38*	*Mi_A_CNL39*	0.0547	0.0514	1.064202335	1.713333333	Tandem
*Mi_A_CNL40*	*Mi_A_CNL41*	0.0793	0.1204	0.658637874	4.013333333	Tandem
*Mi_A_CNL42*	*Mi_A_CNL43*	0.0439	0.0442	0.99321267	1.473333333	Tandem
*Mi_A_CNL42*	*Mi_A_CNL44*	0.0391	0.0307	1.273615635	1.023333333	Tandem
*Mi_A_CNL43*	*Mi_A_CNL44*	0.0335	0.0364	0.92032967	1.213333333	Tandem
*Mi_A_CNL46*	*Mi_A_CNL47*	0.1482	0.1835	0.807629428	6.116666667	Tandem
*Mi_H_CNL4*	*Mi_H_CNL6*	1.4782	1.3332	0.901907726	49.27333333	Tandem
*Mi_H_CNL5*	*Mi_H_CNL6*	1.1388	1.2572	1.10396909	37.96	Tandem
*Mi_H_CNL8*	*Mi_H_CNL9*	1.4903	1.2644	0.848419781	49.67666667	Tandem
*Mi_H_CNL10*	*Mi_H_CNL16*	1.1866	1.2678	1.068430811	39.55333333	Segmental
*Mi_H_CNL18*	*Mi_H_CNL19*	0.0887	0.0526	0.593010147	2.956666667	Tandem
*Mi_H_CNL18*	*Mi_H_CNL20*	1.044	0.8711	0.834386973	34.8	Tandem
*Mi_H_CNL19*	*Mi_H_CNL20*	1.1865	0.8886	0.748925411	39.55	Tandem
*Mi_H_CNL21*	*Mi_H_CNL22*	1.193	1.7234	1.444593462	39.76666667	Tandem
*Mi_T_CNL2*	*Mi_T_CNL23*	0.773	1.054	0.733396584	35.13333333	Segmental
*Mi_T_CNL10*	*Mi_T_CNL17*	3.253	0.2735	11.89396709	9.116666667	Segmental
*Mi_T_CNL11*	*Mi_T_CNL16*	1.0082	0.5716	1.763820854	19.05333333	Segmental
*Mi_T_CNL11*	*Mi_T_CNL17*	1.0941	0.6284	1.741088479	20.94666667	Segmental
*Mi_T_CNL29*	*Mi_T_CNL31*	0.9026	1.4212	0.635097101	47.37333333	Tandem

To analyze the evolutionary constraints of the repeated *MiCNL* genes, the Ka, Ks, and Ka/Ks ratios of all para-homologous gene pairs were also calculated. In *Mi_A_CNLs, Mi_H_CNLs*, and *Mi_T_CNLs* gene pairs had Ka/Ks values ranging from 0.51 to 1.27, 0.59 to 1.44, and 0.63 to 11.89 respectively. Resultantly, the time of divergence of all 50 duplicated gene pairs of *Mi_CNLs* was between 0.3 to 88.4 million years (MYA).

### 3.6 Prediction of *cis*-regulatory elements in the promoter of *MiCNL* genes

**Figure 7:**
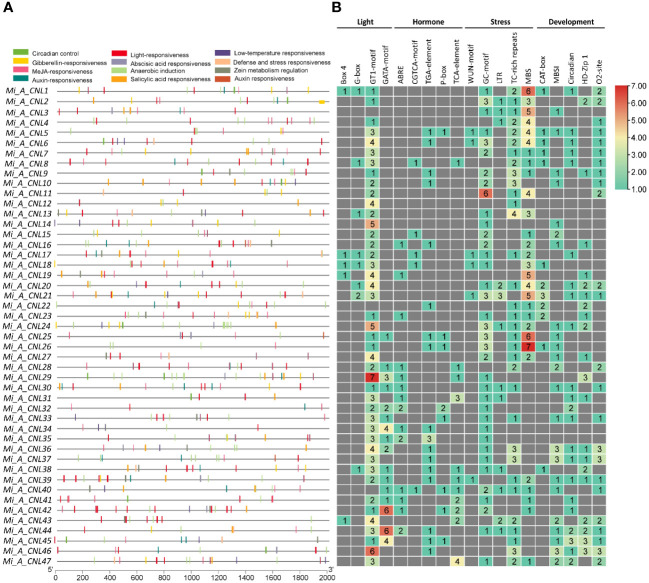
*Cis*-regulatory elements in the promoter region of *Mi_A_CNL* genes. **(A)** Represents the *cis*-elements and their location on the upstream region of genes. **(B)** Represents the heatmap with colors showing the number of elements related to various stresses.

The *cis*-regulatory elements were analyzed to further predict the involvement of *MiCNL* genes in the regulation of abiotic stresses. In all *M. indica* cultivars several *cis*-elements were found which were further classified into light-related, hormone-related, stress-related, and development-related elements (**Table S4**). Regarding these elements, for *cis*-elements Box 4, G-box, GT1-motif, and GATA-motif were found to be involved in light-stress regulation. Five *cis-*elements were involved with hormone responsiveness: ABRE, CGTCA-motif, TGA-element, P-box, and TCA-element. Further, four *cis-*elements were found to be involved with stress responsiveness: GC-motif, LTR, TC-rich repeats, and MBS. Five elements including CAT-box, MBSI, circadian, HD-Zip 1, and o2-site were involved in developmental processes. In *Mi_A_CNLs* light and stress-related *cis*-elements were mostly present (**Figure 7**) while in *Mi_H_CNLs* hormones-related *cis-*elements were mostly present (**Figure S5**). *Mi_T_CNLs* have mostly *cis-*elements related to hormones, stress, and development (**Figure S6**).

### 3.7 PPI and gene ontology enrichment analysis

**Figure 8:**
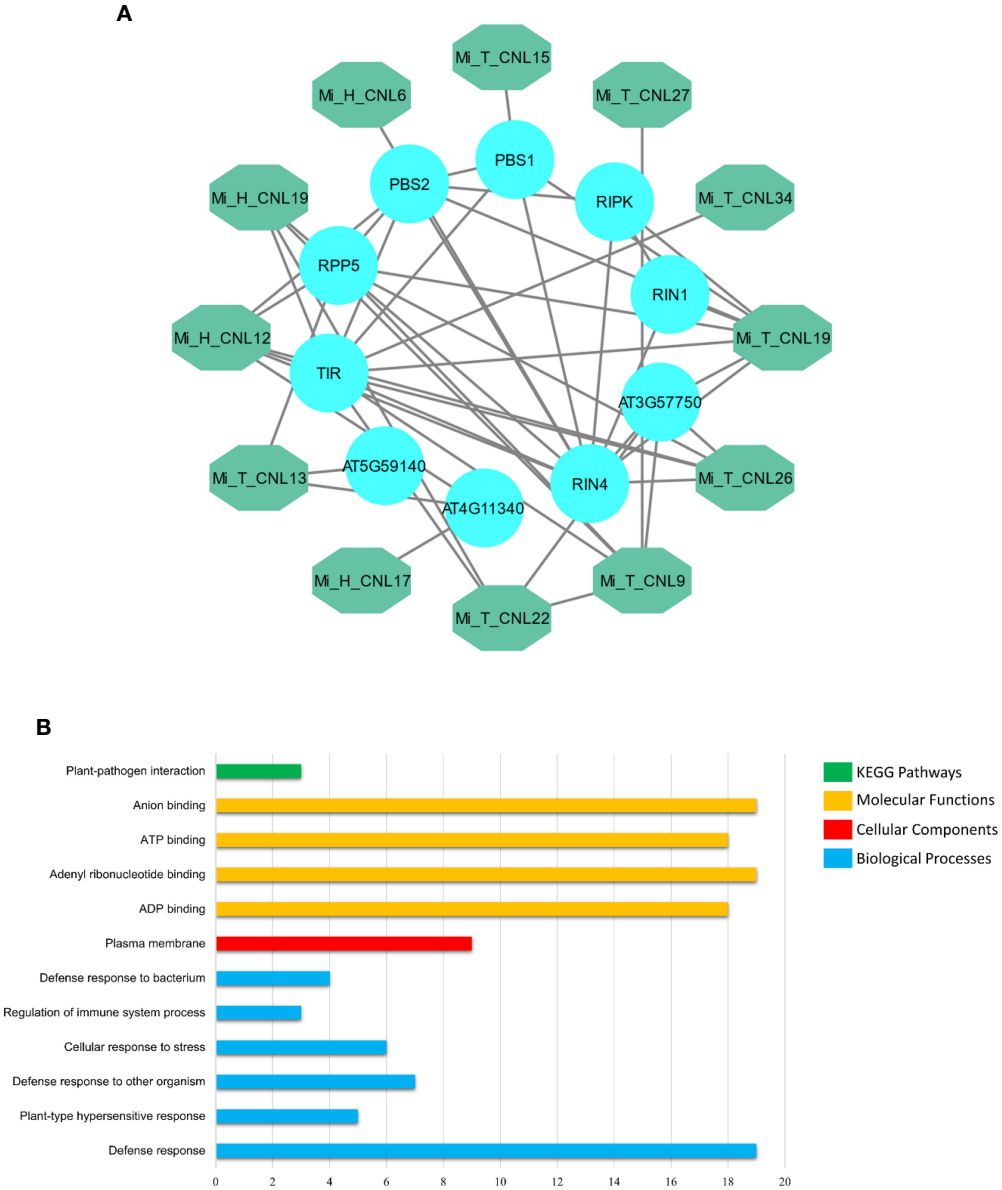
**(A)** Interactions among Mi_A_CNL and other homologous proteins. The teal color represents the Mi_A_CNL proteins and the cyan color represents the other interacting proteins from different species. **(B)** Predicted KEGG pathways, Molecular functions, Cellular components, and Biological Processes associated with Mi_A_CNL proteins.

MiCNL proteins were evaluated to identify interactions among them to understand their functional interactions. Interacting proteins might be involved in a pathway, thus affecting the roles of other proteins and giving an overall response. Some MiCNL proteins were found to interact with the other CNL as well as the other homologous proteins. Among MiCNLs, Mi_T_CNLs showed the highest interactions. Mi_T_CNL18 and Mi_T_CNL12 were among the highly interacting proteins. Further, Mi_T_CNL9, Mi_T_CNL26, and Mi_H_CNL2 also showed great interactions with other defense-responsive proteins (**Figure 8A**).

Gene Ontology (GO) enrichment analysis was performed on the *MiCNL* genes. According to GO analysis, these genes were involved in a KEGG pathway: Plant pathogen interactions (GO: ath04626), Molecular functions including ADP binding (GO:0043531), Adenyl ribonucleotide binding (GO:0032559), ATP binding (GO:0005524) and Anion binding (GO:0043168). Moreover, these proteins were found to be in the plasma membrane (GO:0005886). These proteins also participate in a variety of biological processes including Defense response (GO:0006952), Plant-type hypersensitive response (GO:0009626), Defense response to other organisms (GO:0098542), Cellular response to stress (GO:0033554), Regulation of immune system process (GO:0002682), and Defense response to the bacterium (GO:0042742) (**Table S5**; **Figure 8B**).

### 3.8 Expression analysis of *Mi_A_CNL* genes

**Figure 9:**
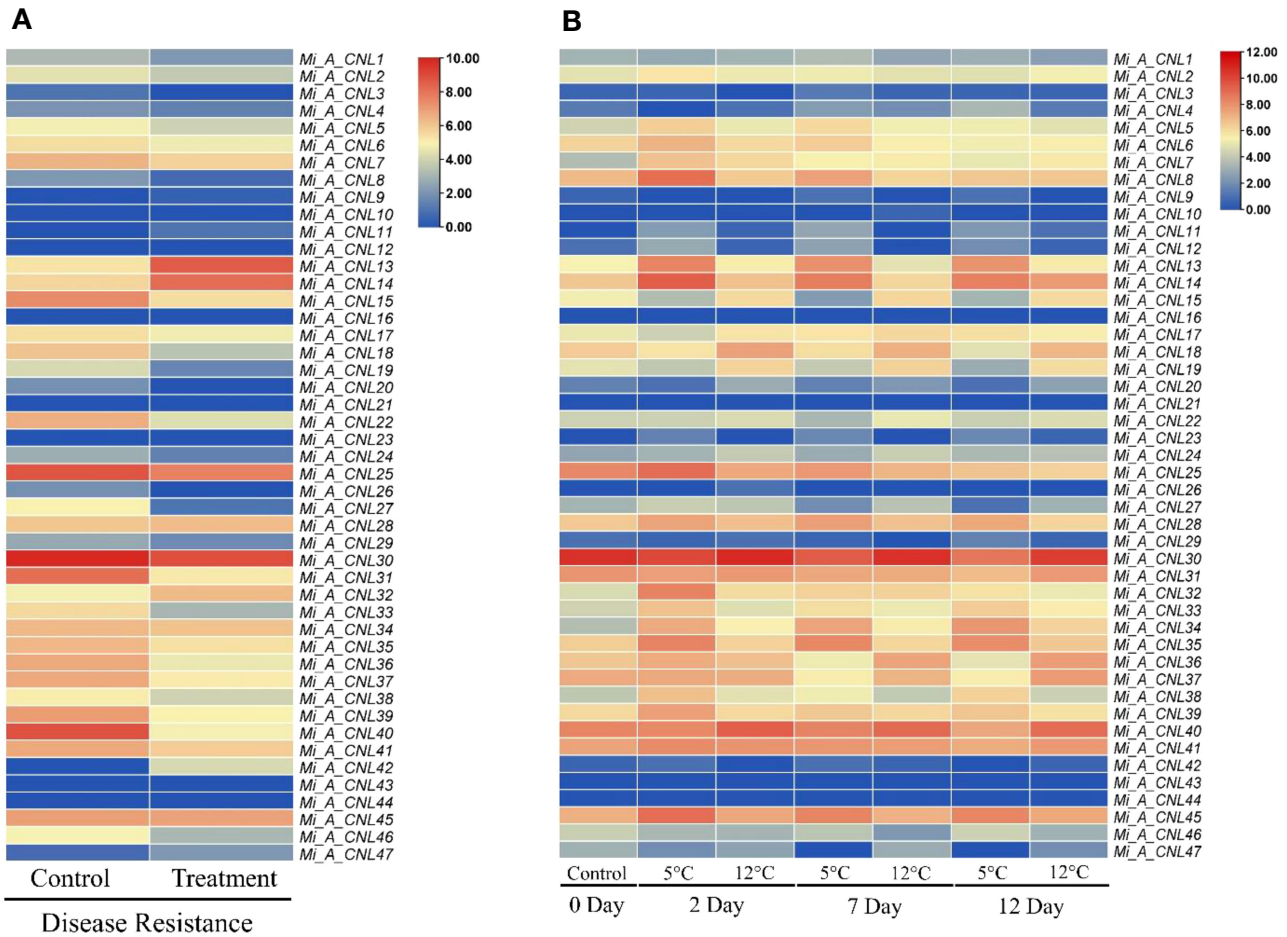
Heatmap regarding the expression pattern of Mi_A_*CNL* genes in fruit peel at different conditions constructed using count values. **(A)** Disease stress **(B)** Cold stress at 2, 7, and 12 days. The red color represents the up-regulated expression and the blue color represents the higher or upregulated expression.

To further investigate the roles of these genes, their expression patterns were observed in disease and cold stress. In the disease stage, few genes showed fluctuated expression as *Mi_A_CNL13* and *Mi_A_CNL14* were up-regulated in fruit peel and *Mi_A_CNL15, 25, 30, 31, 40* were down-regulated (**Figure 9A**). In cold stress *Mi_A_CNL2, 14, 41, 45* were up-regulated and *Mi_A_CNL47* is down-regulated (**Figure 9B**). Overall, the expression level of the remaining genes was found to be similar in each stress and condition.

### 3.9 Structure prediction of Mi_A_CNL proteins

**Figure 10:**
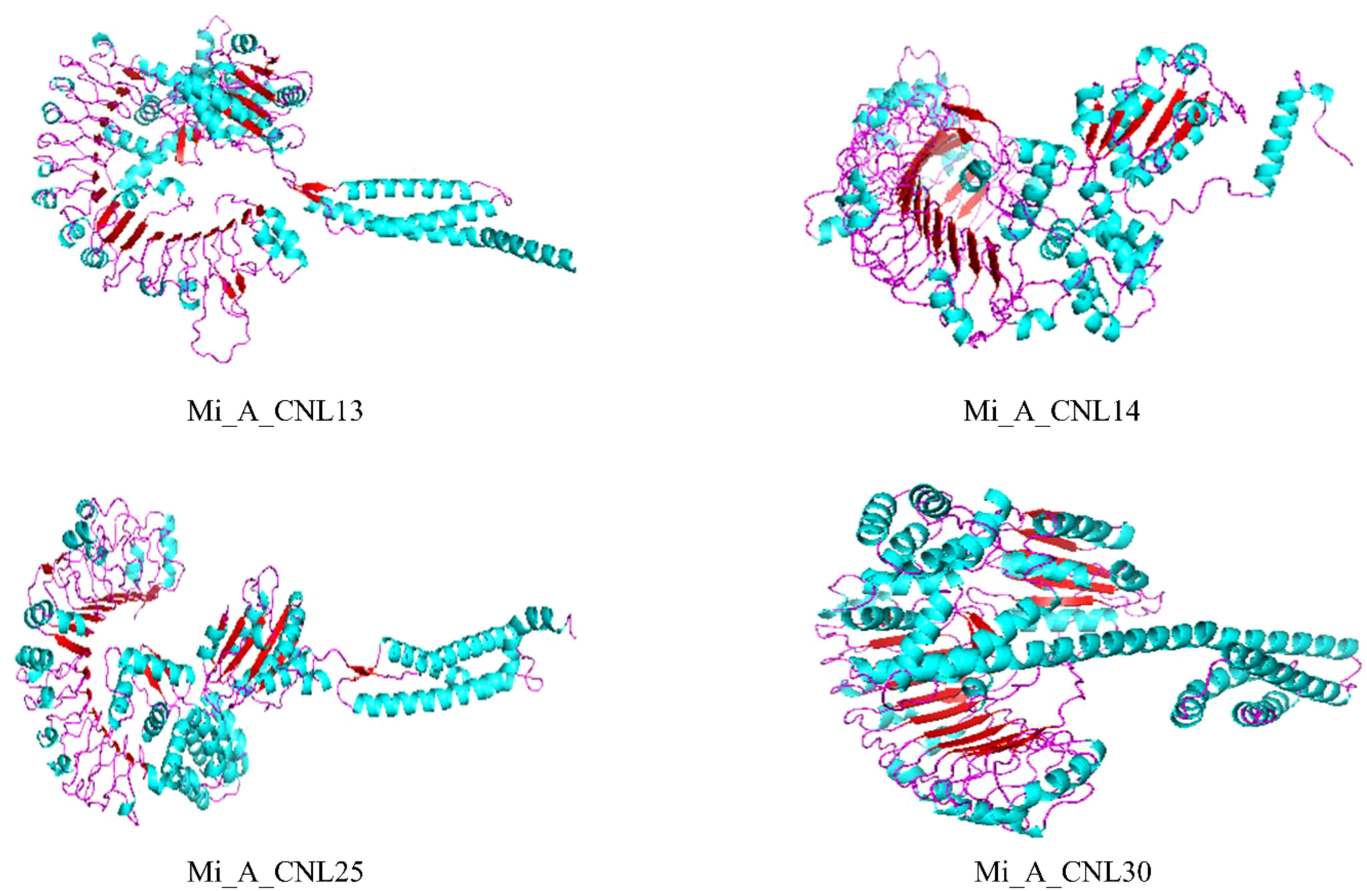
Predicted 3D structures of four Mi_A_CNLs using Alphaflold2 and visualized using PyMOL. Red color represents the helices, cyan color represents the sheets, and pink color represents the loops.

To obtain more structural and ultimately functional insights, the 3D structures of four Mi_A_CNLs proteins (Mi_A_CNL13, 14, 25, and 30) were modeled. All these structures shared almost similar structures of loops, helices, and turns. All these structures contained a great number of helices. The basic structure was similar such as turns on the left side of structures (leucine-rich repeats) are visible in every modeled protein. Moreover, the number of helices in each protein is also the same (**Figure 10**).

### 3.10 Performance evaluation of multi-stress responsive genes

A total of 15 genes were found to be present in both disease and cold datasets. A machine learning classifier, a random forest algorithm, was employed to assess their performance (**Table 3**). Using the count’s data of disease stress as the training dataset, it was analyzed that only one gene (*Mi_A_CNL14)* was rigorously tested for its multi-stress responsiveness. The classification model’s sensitivity, specificity, and overall accuracy were evaluated using the Receiver Operating Characteristic (ROC) plot. Impressively, *Mi_A_CNL14* demonstrated a ROC value of 0.8333, indicating its acceptable performance as a potential multi-stress responsive gene. **Figure S7** visually represents the ROC plot for *Mi_A_CNL14*, providing supporting evidence of its classification efficacy.

**Table 3:** Summary of common DEGs identified in disease and cold stress.

Gene Symbol	Disease		Cold	
	log2FC ( ± 0.5)	p-value (<0.05)	log2FC ( ± 0.5)	p-value (<0.05)
*Mi_A_CNL13*	-4.7026	7.13E-176	2.552667	1.32E-23
*Mi_A_CNL14*	-3.91602	4.2E-115	0.932222	2.63E-08
*Mi_A_CNL15*	0.638674	0.001414	-2.4964	2.6E-08
*Mi_A_CNL18*	1.332636	0.000346	-1.92957	3.33E-11
*Mi_A_CNL19*	1.716102	0.027093	-2.79916	2.25E-09
*Mi_A_CNL27*	3.434271	0.000113	-1.95296	0.01815
*Mi_A_CNL28*	-1.55853	7.4E-13	1.4328	4.57E-09
*Mi_A_CNL31*	1.611888	1.22E-16	-0.68161	0.000286
*Mi_A_CNL32*	-2.81224	1.91E-27	1.02666	0.00068
*Mi_A_CNL33*	1.305953	0.003225	1.104997	0.000268
*Mi_A_CNL34*	-1.07779	1.38E-07	1.855967	1.68E-14
*Mi_A_CNL36*	0.818557	0.002012	-2.52027	2.35E-20
*Mi_A_CNL39*	0.73157	0.001935	1.01412	5.69E-05
*Mi_A_CNL40*	2.679719	1.49E-43	-1.31204	9.77E-20
*Mi_A_CNL45*	-1.24695	9.26E-14	1.117963	2.01E-11

## 4 Discussion

Disease resistance R genes in plants are essential for effector-triggered immunity (ETI) because they have mechanisms for identifying pathogens in plants and protecting the plants directly or indirectly (Yang and Wang, 2015). The NBS-LRR class of these R genes, having most of the NBS and LRR domains at the C-terminal, encodes the largest family of all the five classes of these proteins (Meyers et al., 2003). Two major subfamilies of the NBS-LRR protein family are usually found: toll/interleukin-1 receptor-NBS-LRR (TNL) and coil-coil-NBS-LRR (CNL) (Shao et al., 2014).

In this study, three mango cultivars were analyzed to identify CNL genes in their genome by constructing a draft pan-genome. The CNL gene family is widespread in a variety of plant species such as *C. sinensis* (Yin et al., 2023), *Carica papaya* (Porter et al., 2009), *C. sativus L*. (Zhang et al., 2022), *H. annuus L*. (Neupane et al., 2018), *O. sativa* (Zhou et al., 2004), *Populus trichocarpa* (Kohler et al., 2008), *Solanum lycopersicum* (Andolfo et al., 2013), *Solanum tuberosum* (Jupe et al., 2012), and *B. rapa* (Liu et al., 2021). In this study 47, 27, and 36 CNL genes were found in *Alphonso, H. Xiang Ya, and T. atkins* respectively. The varying number of CC-NBS-LRR genes, specifically *MiCNL* genes, among three mango cultivars (ranging from 27 to 47) suggests intraspecific genomic diversity. This phenomenon may be attributed to factors such as genetic drift, environmental pressures, historical hybridization events, gene duplications, and transposon-mediated processes. Similarly, these members showed greater variation in numbers among different plants such as *B. rapa* which has 40 members [61]. The radish genome had 19 members [55]. Furthermore, *A. thaliana* has 51 identified members (Meyers et al., 2003).

Pan-genome wide analysis provides a comprehensive overview of diversity at the genomic level involving multiple species, which may lead to the identification of unique genes that are present in specific species instead of being present in all genomes under study (Tahir ul Qamar et al., 2020). Similarly, in this study, three unique genes were identified only in the *H. Xiang Ya* cultivar including *Mi_H_CNL3, 12*, and *13*. The phylogenetic analysis categorized CNL genes into four groups (A, B, C, and D) using *A. thaliana* as a reference. The clade of Group C was the largest and Group D was the smallest. All of these genes belonging to the same subgroup were clustered together and shared the same homology, even members from the other species as well. However, none of the CNL genes from *Alphonso* and *T. atkins* were found in group D. Similarly, in the case of *C. sativus L*. no gene was present in group D (Zhang et al., 2022). *H. Xiang Ya* is the only cultivar that has three members in group D named *Mi_H_CNL3, 12*, and *13*.

The conservation of motifs and gene structures was similar to the ones observed in previous studies like *C. sinensis* and *B. rapa*, in which very few such as one exon or intron were found. Similarly, the conservation of motifs among groups was also the same (Kohler et al., 2008). The observed differences in the number of exons and introns among mango cultivars and other species imply the evolutionary changes in gene structures over time, potentially impacting their functional conservation. This suggests diversification of CNL genes. Despite this variation in gene structure, most genes share a similar number of conserved motifs, indicating the preservation of their functions throughout evolution.

Chromosomal mapping indicates that the CNL genes in all three cultivars are distributed unevenly but among all cultivars, most of the genes are present in the form of clusters. The same trend was observed in *A. thaliana* (Meyers et al., 2003), *C. sinensis* (Yin et al., 2023), *R. sativus L.* (Ma et al., 2021), *B. rapa* (Liu et al., 2021), and *O. sativa* (Zhou et al., 2004). Most of these genes in three cultivars were found to have undergone tandem duplication. A similar pattern of duplication was observed in *B. rapa* (Ma et al., 2021) and *C. sinensis* (Yin et al., 2023). The evaluation of selection pressure on genes involved the use of the Ka/Ks ratio, which represents the ratio of non-synonymous (Ka) to synonymous (Ks) mutations. A Ka/Ks ratio greater than 1 indicates positive selection, while a ratio less than 1 signifies purifying selection. The analysis of mango cultivars revealed evidence of both positive and purifying selection acting on the studied genes.

The promoter region of these genes showed several stress-related elements that further confirm the involvement of these genes in different abiotic and disease-resistant stresses. Other plants have also been shown to have these elements which confer resistance to various environmental stresses. Black rot (BR) is a bacterial disease caused by *Xanthomonas campestris pv. campestris dowson*, which infects many *Brassica* species, such as cabbage (*Brassica oleracea var*), Chinese cabbage (*Brassica pekinensis*), and oil seed rape (*Brassica campestris)* (Zeilmaker et al., 2015; Zhang et al., 2018). All these findings help us understand the involvement of these genes in various stresses.

Protein-Protein interaction studies showed that few proteins from mango cultivars interacted with other defense-responsive proteins including TIR, RIN1, RIN4, PBS1, PBS2, and RPP5. GO analysis revealed that most CNL genes are located in the plasma membrane and involved in defense responses, ADP binding, ATP binding, anion binding, and adenyl ribonucleotide binding. In *Vitis vinifera*, NBS-LRR genes are also involved in defense responses, ADP binding, and ATP binding (Goyal et al., 2020).

The expression profiling of these genes showed their varied expression in disease and cold stress. The expression was analyzed in fruit peel. In response to disease stress, *Mi_A_CNL13* and *Mi_A_CNL14* were up-regulated, whereas *Mi_A_CNL15, 25, 30, 31*, and *40* were down-regulated. Conversely, under cold stress, *Mi_A_CNL2, 14, 41*, and *45* were up-regulated, while *Mi_A_CNL47* was down-regulated. In *B. rapa* most of the CNL genes have the same trend but in *C. sativus L.* most of the CNL genes were up-regulated in salt and chilling (cold) stress (Liu et al., 2021; Zhang et al., 2022). Based on expression values the 3D structures of four proteins were also predicted to help understand their structural as well as functional conservations and all four proteins have almost the same number of alpha-helices and beta sheets.

Furthermore, random forest, the machine learning approach was utilized to evaluate the genes that were showing multi-stress responses in both disease and cold stress. A total of 15 genes were common in both datasets but only one gene (*Mi_A_CNL14*) was significantly involved in multi-stress response. Some other studies also utilized the same methods to evaluate genes involved in multi-stress response (Fatima et al., 2023). Therefore, it can be concluded that CNL genes can significantly benefit mango genetic improvement through breeding or genetic manipulation, by conferring disease resistance and enhancing tolerance to abiotic stresses. Their role in multi-stress responsiveness, as suggested by our analysis, makes them valuable candidates for further breeding programs seeking mango varieties with robust adaptability to diverse environmental conditions. Breeding for MiCNL gene related traits could lead to healthier mango plants, reduced pesticide dependency, and improved sustainability in mango cultivation.

## 5 Conclusion

In this study, a draft pan-genome was constructed and PAVs were scanned through the ppsPCP pipeline using three mango cultivars in which 47 genes in *Alphonso*, 27 in *H. Xiang Ya*, and 36 in *T. atkins* have been identified. These were classified into four groups: A, B, C, and D. All the members from the same group shared greater conservation in motif and gene structure. Few segmental and most tandemly duplicated pairs were found. A large number of *cis-*regulatory elements related to light, hormones, stress, and development responsive were found in promoter regions of mango *CNLs*. PPI showed CNL proteins interact with CNL and other defense-responsive proteins. and GO enrichment analysis revealed their interaction and involvement in pathways as well as processes related to defense response. Structure prediction showed high similarity among members of the same groups. Expression profiling of mango fruit peel under disease stress revealed that *Mi_A_CNL13* and *14* were up-regulated while *Mi_A_CNL15, 25, 30, 31, and 40 were* down-regulated. On the other hand, in cold stress *Mi_A_CNL2, 14, 41, 45* were up-regulated and *Mi_A_CNL47* is down-regulated. Machine learning approaches indicate that out of 15 common genes, only one gene (*Mi_A_CNL14*) can be a multi-stress responsive gene (Super gene). Our results provide a solid foundation to further investigate the function of *CNLs* in regulating various abiotic and environmental stress responses and more accessions should be sequenced to improve the quality of the reference genome.

## Data availability statement

The original contributions presented in the study are included in the article/**Supplementary Material**. Further inquiries can be directed to the corresponding authors.

## Author contributions

MT: Conceptualization, Data curation, Formal Analysis, Funding acquisition, Investigation, Methodology, Project administration, Software, Visualization, Writing – original draft. MS: Data curation, Investigation, Methodology, Visualization, Writing – original draft. X-TZ: Data curation, Methodology, Software, Validation, Writing – review & editing. HL: Data curation, Investigation, Methodology, Validation, Writing – review & editing. XH: Conceptualization, Data curation, Methodology, Validation, Visualization, Writing – review & editing. KF: Data curation, Investigation, Methodology, Validation, Writing – review & editing. MA: Data curation, Formal analysis, Funding acquisition, Investigation, Resources, Validation, Writing – review & editing. L-LC: Conceptualization, Funding acquisition, Methodology, Project administration, Resources, Supervision, Validation, Writing – review & editing.
