# Supplementary figures and images for: Comparative genomics profiling revealed multi-stress responsive roles of the CC-NBS-LRR genes in three mango cultivars
Source: Front Plant Sci. 2023 Oct 30;14:1285547. doi: 10.3389/fpls.2023.1285547 (PMC10642748; doi:10.3389/fpls.2023.1285547)

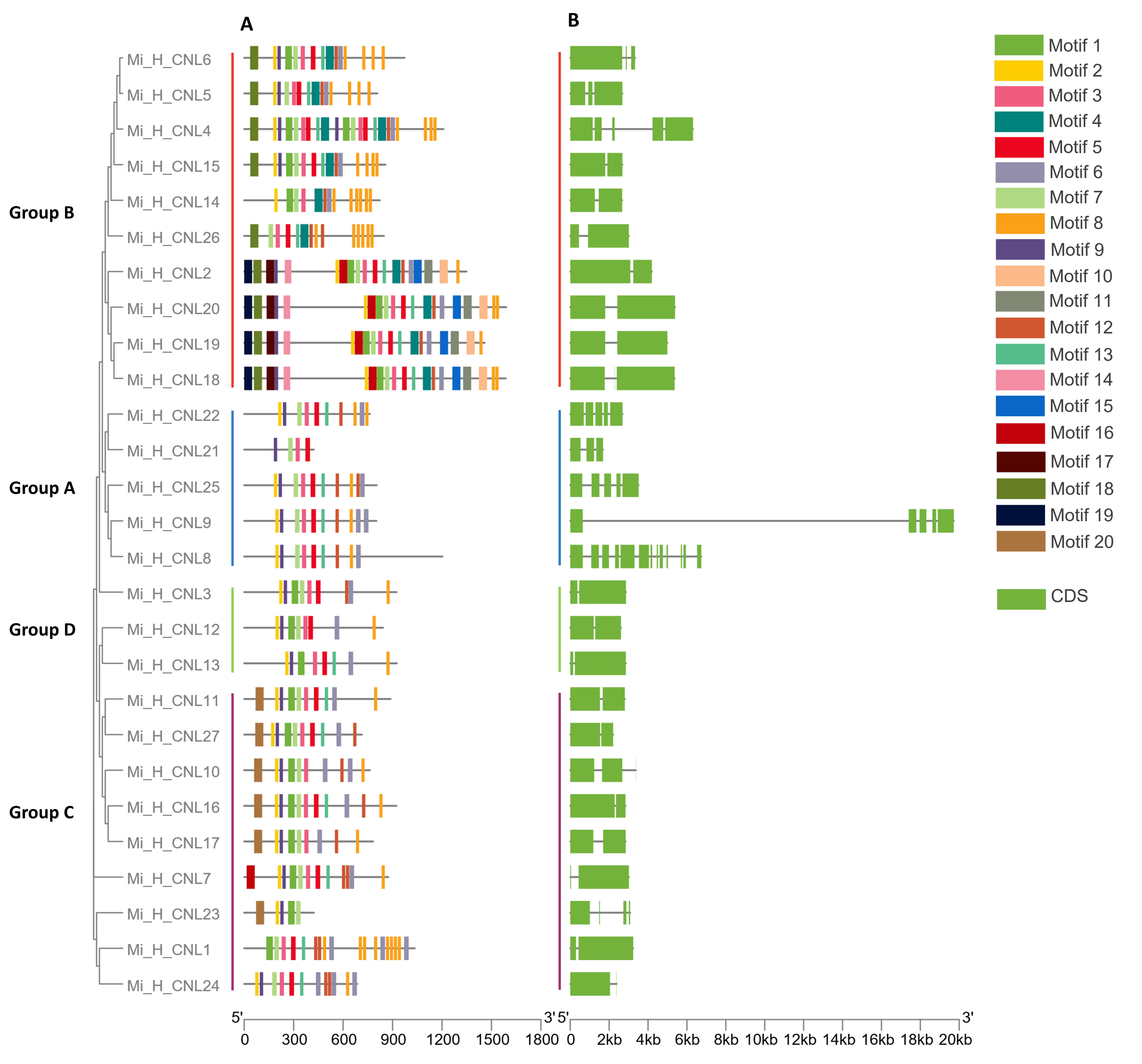

Supplement: Supplementary file 1 [file Image_1.tif]

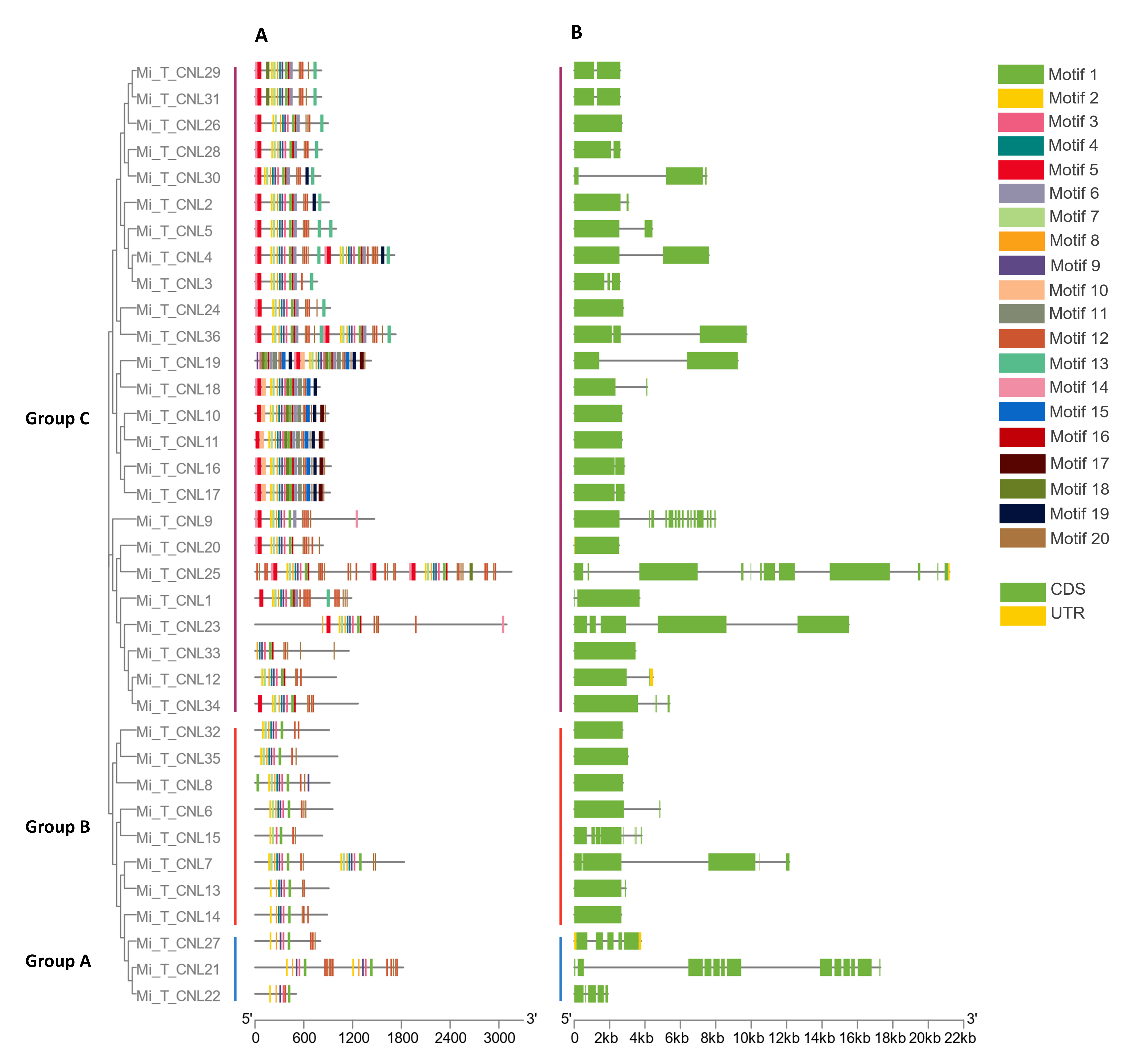

Supplement: Supplementary file 2 [file Image_2.tif]

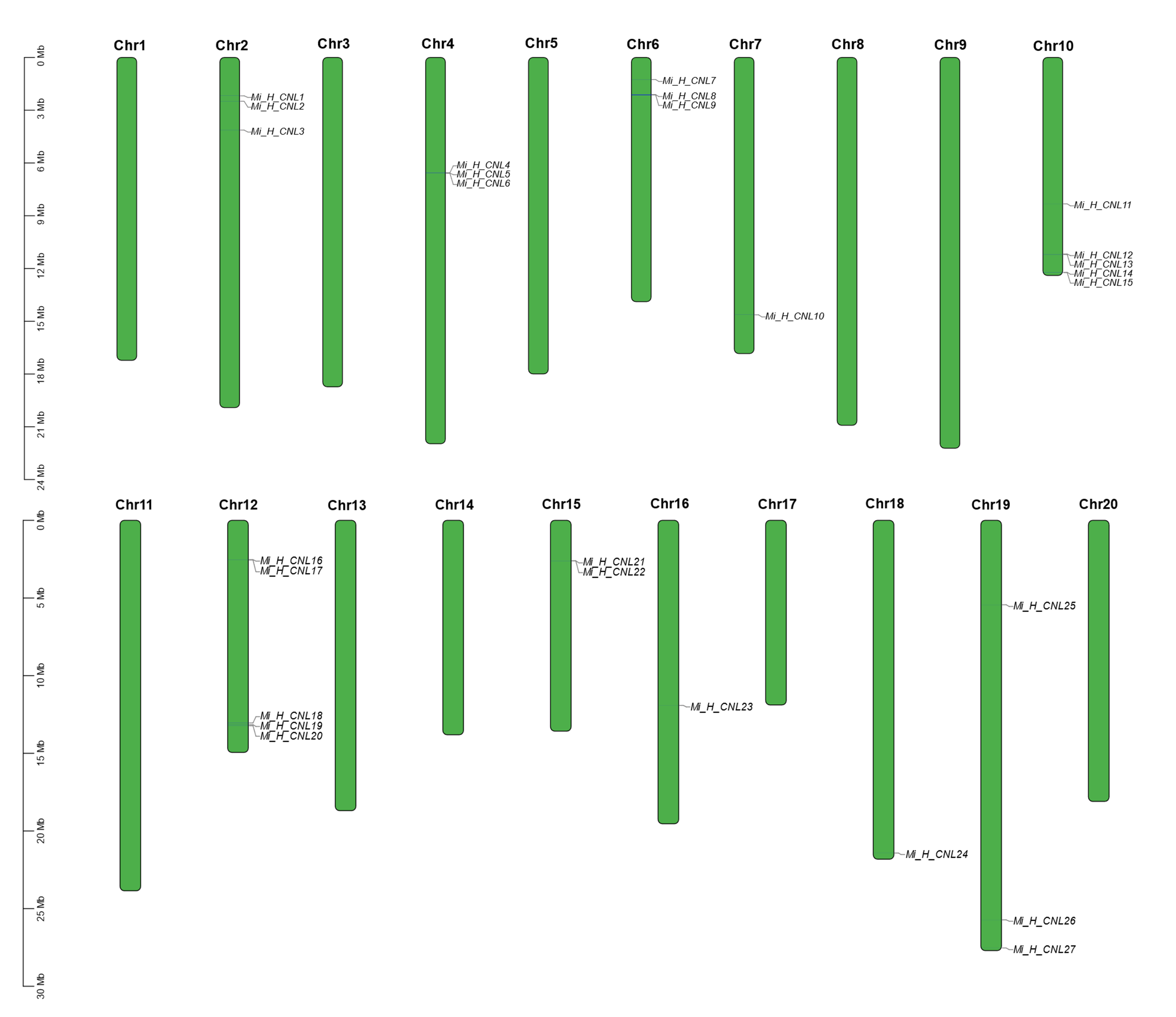

Supplement: Supplementary file 3 [file Image_3.tif]

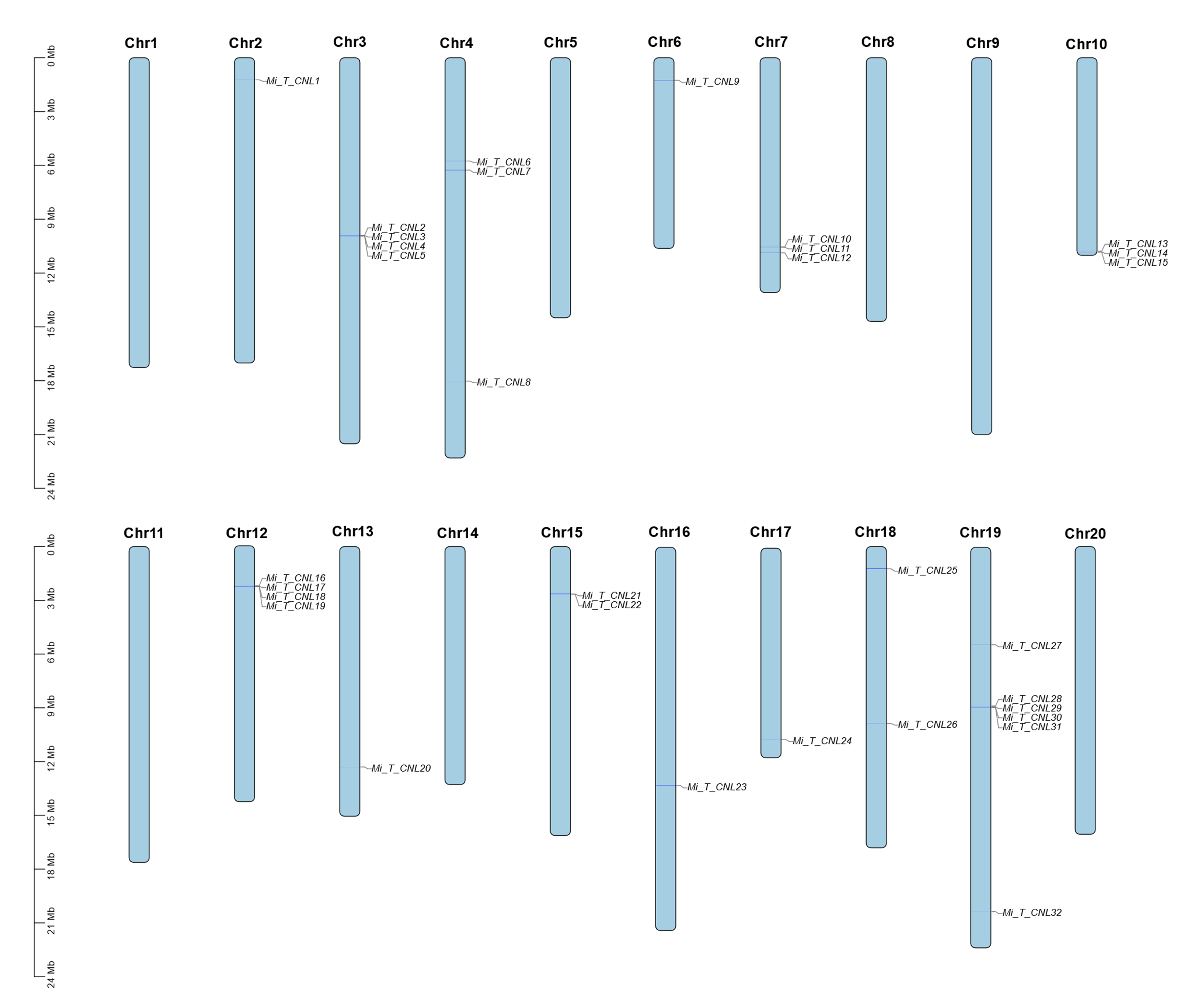

Supplement: Supplementary file 4 [file Image_4.tif]

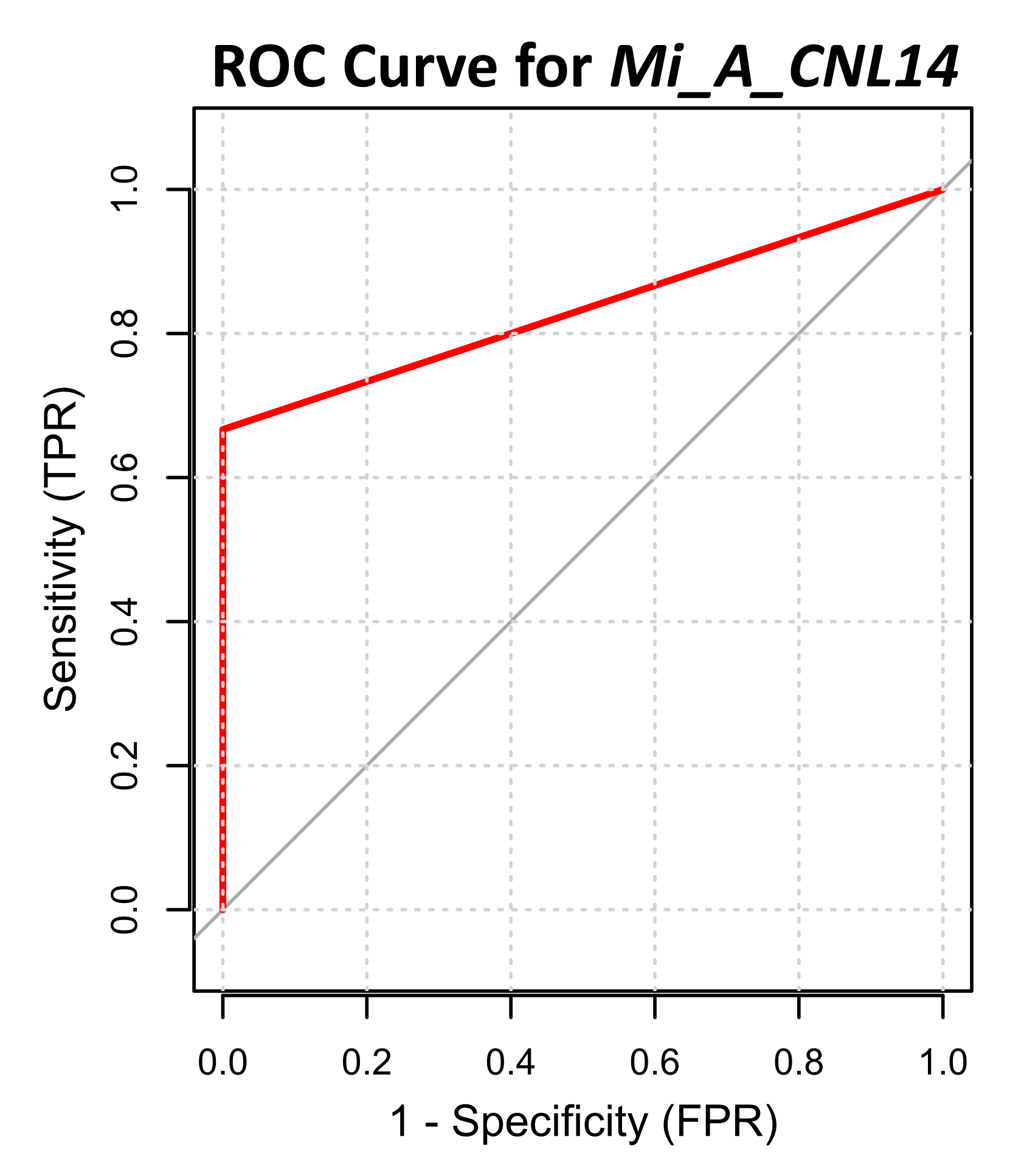

Supplement: Supplementary file 7 [file Image_7.tif]
