# Supplementary material for: Comparative genomics profiling revealed multi-stress responsive roles of the CC-NBS-LRR genes in three mango cultivars
Source: Front Plant Sci. 2023 Oct 30;14:1285547. doi: 10.3389/fpls.2023.1285547 (PMC10642748; doi:10.3389/fpls.2023.1285547)
Supplement: Supplementary file 9 [file Table_2.docx]

| Gene Name | Pfam | HMMER | CDD | Interpro |
| --- | --- | --- | --- | --- |
| Mi_A_CNL1 | RPW8, NB-ARC | RPW8, NB-ARC | RPW8 superfamily, NB-ARC superfamily, PLN03210 superfamily | LRR_dom_sf, NB-ARC, P-loop_NTPase, Powdery_mildew-R_dom, Powdery_mildew-R_dom, Apaf_helical, RPW8-like, WH-like_DNA-bd_sf |
| Mi_A_CNL2 | RPW8, NB-ARC | RPW8, NB-ARC | RPW8 superfamily, NB-ARC superfamily, PLN03210 superfamily, LRR superfamily | Powdery_mildew-R_dom, RPW8-like, P-loop_NTPase, Apaf_helical, WH-like_DNA-bd_sf, LRR_dom_sf, NB-ARC |
| Mi_A_CNL3 | NB-ARC, Rx_N, LRR_8 | Rx_N, NB-ARC | NB-ARC superfamily, RX-CC_like, LRR superfamily | RX-like_CC, Rx_N, NB-ARC, WH-like_DNA-bd_sf, LRR_dom_sf, Disease_R_plants, P-loop_NTPase, Apaf_helical |
| Mi_A_CNL4 | NB-ARC, Rx_N, LRR_8 | Rx_N, NB-ARC | NB-ARC superfamily, RX-CC_like, PLN00113 superfamily | P-loop_NTPase, WH-like_DNA-bd_sf, Disease_R_plants, RX-like_CC, NB-ARC, Rx_N, LRR_dom_sf, Apaf_helical |
| Mi_A_CNL5 | NB-ARC, LRR_8 | NB-ARC, LRR_8 | NB-ARC superfamily, PLN03210 superfamily | Leu-rich_rpt, LRR_dom_sf, Apaf_helical, NB-ARC, Leu-rich_rpt_typical-subtyp, P-loop_NTPase, WH-like_DNA-bd_sf |
| Mi_A_CNL6 | NB-ARC, Rx_N, LRR_8 | Rx_N, NB-ARC, LRR_8 | NB-ARC superfamily, Rx_N, PLN03210 superfamily, PLN03210 superfamily | P-loop_NTPase, Rx_N, Leu-rich_rpt, NB-ARC, LRR_dom_sf, Disease_R_plants, Apaf_helical, WH-like_DNA-bd_sf |
| Mi_A_CNL7 | NB-ARC, Rx_N, LRR_8 | Rx_N, NB-ARC | NB-ARC superfamily, Rx_N, PLN03210 superfamily, PLN03210 superfamily, PLN03210 superfamily | WH-like_DNA-bd_sf, Rx_N, Disease_R_plants, NB-ARC, P-loop_NTPase, LRR_dom_sf, Apaf_helical |
| Mi_A_CNL8 | NB-ARC, Rx_N, LRR_8 | Rx_N, NB-ARC | NB-ARC superfamily, RX-CC_like, PLN00113 superfamily | LRR_dom_sf, P-loop_NTPase, WH-like_DNA-bd_sf, Disease_R_plants, RX-like_CC, Apaf_helical, NB-ARC, Rx_N |
| Mi_A_CNL9 | NB-ARC, Rx_N | Rx_N, NB-ARC | NB-ARC superfamily, RX-CC_like | NB-ARC, Rx_N, LRR_dom_sf, Disease_R_plants, P-loop_NTPase, WH-like_DNA-bd_sf, Apaf_helical, RX-like_CC |
| Mi_A_CNL10 | NB-ARC, Rx_N, LRR_8 | Rx_N, NB-ARC | NB-ARC superfamily, RX-CC_like, LRR superfamily | RX-like_CC, Rx_N, Apaf_helical, Disease_R_plants, NB-ARC, P-loop_NTPase, WH-like_DNA-bd_sf, LRR_dom_sf |
| Mi_A_CNL11 | NB-ARC, Rx_N, LRR_4 | Rx_N, NB-ARC | NB-ARC superfamily, RX-CC_like, LRR superfamily | Disease_R_plants, Apaf_helical, WH-like_DNA-bd_sf, P-loop_NTPase, NB-ARC, RX-like_CC, Rx_N, LRR_dom_sf |
| Mi_A_CNL12 | RPW8, NB-ARC | Rx_N, NB-ARC | NB-ARC superfamily, RX-CC_like, PLN03210 superfamily | P-loop_NTPase, Rx_N, Apaf_helical, RX-like_CC, LRR_dom_sf, NB-ARC, WH-like_DNA-bd_sf, Disease_R_plants |
| Mi_A_CNL13 | RPW8, NB-ARC | NB-ARC | NB-ARC superfamily, LRR superfamily, PLN03210 superfamily, PLN03210 superfamily | P-loop_NTPase, Apaf_helical, AAA+_ATPase, NB-ARC, LRR_dom_sf |
| Mi_A_CNL14 | NB-ARC, Rx_N, LRR_8 | NB-ARC | NB-ARC superfamily, LRR superfamily, PLN03210 superfamily | LRR_dom_sf, P-loop_NTPase, Apaf_helical, NB-ARC |
| Mi_A_CNL15 | NB-ARC, Rx_N, LRR_8 | NB-ARC, LRR_8 | NB-ARC superfamily, LRR, | Leu-rich_rpt, P-loop_NTPase, LRR_dom_sf, Apaf_helical, Leu-rich_rpt_typical-subtyp, NB-ARC, WH-like_DNA-bd_sf |
| Mi_A_CNL16 | NB-ARC, LRR_8 | NB-ARC, LRR_8 | NB-ARC superfamily, LRR | Leu-rich_rpt, P-loop_NTPase, Leu-rich_rpt_typical-subtyp, LRR_dom_sf, WH-like_DNA-bd_sf, Apaf_helical, NB-ARC |
| Mi_A_CNL17 | NB-ARC, Rx_N, LRR_8 | NB-ARC, LRR_8 | NB-ARC, LRR_8,  PLN00113 superfamily, bZIP superfamily, FlhF superfamily | NB-ARC, P-loop_NTPase, LRR_dom_sf, WH-like_DNA-bd_sf, Leu-rich_rpt, Apaf_helical |
| Mi_A_CNL18 | NB-ARC, Rx_N, LRR_8 | NB-ARC, LRR_8 | NB-ARC superfamily, LRR | P-loop_NTPase, WH-like_DNA-bd_sf, LRR_dom_sf, NB-ARC, Leu-rich_rpt, Apaf_helical |
| Mi_A_CNL19 | NB-ARC, Rx_N, LRR_8 | NB-ARC, LRR_8 | NB-ARC, LRR, bZIP superfamily | LRR_dom_sf, Apaf_helical, P-loop_NTPase, WH-like_DNA-bd_sf, NB-ARC, Leu-rich_rpt |
| Mi_A_CNL20 | NB-ARC, Rx_N | NB-ARC, LRR_8 | NB-ARC, PLN03210 superfamily | Leu-rich_rpt_typical-subtyp, Leu-rich_rpt, NB-ARC, Apaf_helical, WH-like_DNA-bd_sf, P-loop_NTPase, AAA+_ATPase, LRR_dom_sf |
| Mi_A_CNL21 | NB-ARC, Rx_N, LRR_8 | NB-ARC, LRR_8 | NB-ARC superfamily, LRR_8, PLN03210 superfamily | WH-like_DNA-bd_sf, Leu-rich_rpt, P-loop_NTPase, AAA+_ATPase, LRR_dom_sf, NB-ARC, Apaf_helical, Growth_fac_rcpt_cys_sf, Leu-rich_rpt_typical-subtyp |
| Mi_A_CNL22 | NB-ARC, Rx_N, LRR_4 | NB-ARC, LRR_8 | NB-ARC superfamily, LRR | Leu-rich_rpt, NB-ARC, WH-like_DNA-bd_sf, LRR_dom_sf, Apaf_helical, P-loop_NTPase |
| Mi_A_CNL23 |  | NB-ARC | NB-ARC superfamily, LRR superfamily | P-loop_NTPase, Apaf_helical, LRR_dom_sf, AAA+_ATPase, NB-ARC |
| Mi_A_CNL24 |  | Rx_N, NB-ARC, LRR_8 | NB-ARC superfamily, PLN03210 superfamily, Rx_N | Apaf_helical, Leu-rich_rpt, LRR_dom_sf, Leu-rich_rpt_typical-subtyp, Rx_N, Disease_R_plants, P-loop_NTPase, WH-like_DNA-bd_sf, NB-ARC |
| Mi_A_CNL25 |  | Rx_N, NB-ARC | NB-ARC superfamily, Rx_N, PLN03210 superfamily, PLN03210 superfamily, PLN03210 superfamily | P-loop_NTPase, LRR_dom_sf, RX-like_CC, Apaf_helical, Disease_R_plants, NB-ARC, Rx_N, WH-like_DNA-bd_sf |
| Mi_A_CNL26 | NB-ARC, Rx_N, LRR_8 | Rx_N, NB-ARC | NB-ARC superfamily, Rx_N, PLN03210 superfamily, PLN00113 superfamily, PLN03210 superfamily | P-loop_NTPase, LRR_dom_sf, Disease_R_plants, Apaf_helical, Leu-rich_rpt_typical-subtyp, Rx_N, NB-ARC, WH-like_DNA-bd_sf |
| Mi_A_CNL27 | NB-ARC, Rx_N, LRR_8 | Rx_N, NB-ARC | NB-ARC superfamily, Rx_N, LRR superfamily | WH-like_DNA-bd_sf, P-loop_NTPase, Disease_R_plants, Rx_N, NB-ARC, LRR_dom_sf, Apaf_helical, RX-like_CC |
| Mi_A_CNL28 | NB-ARC, Rx_N, LRR_8 | Rx_N, NB-ARC, LRR_8 | NB-ARC superfamily, Rx_N, LRR superfamily, | Apaf_helical, WH-like_DNA-bd_sf, P-loop_NTPase, Disease_R_plants, Leu-rich_rpt, NB-ARC, LRR_dom_sf, Rx_N |
| Mi_A_CNL29 | NB-ARC, RPW8, LRR_8 | RPW8, NB-ARC | NB-ARC superfamily, PLN03210 superfamily, RPW8 superfamily | WH-like_DNA-bd_sf, Apaf_helical, P-loop_NTPase, Powdery_mildew-R_dom, RPW8-like, NB-ARC, LRR_dom_sf |
| Mi_A_CNL30 | NB-ARC, RPW8, LRR_8 | RPW8, NB-ARC | NB-ARC superfamily, PLN03210 superfamily, RPW8 superfamily | WH-like_DNA-bd_sf, NB-ARC, P-loop_NTPase, Apaf_helical, Powdery_mildew-R_dom, RPW8-like, LRR_dom_sf |
| Mi_A_CNL31 | RPW8, NB-ARC | RPW8, NB-ARC | NB-ARC superfamily, PLN03210 superfamily, RPW8 superfamily | RPW8-like, Apaf_helical, NB-ARC, Powdery_mildew-R_dom, LRR_dom_sf, [P-loop_NTPase](https://www.ebi.ac.uk/interpro/entry/InterPro/IPR027417/), [WH-like_DNA-bd_sf](https://www.ebi.ac.uk/interpro/entry/InterPro/IPR036388/) |
| Mi_A_CNL32 | RPW8, NB-ARC | Rx_N, NB-ARC, LRR_8 | NB-ARC superfamily, Rx_N, PLN03210 superfamily, PLN03210 superfamily, PLN03210 superfamily | [Rx_N](https://www.ebi.ac.uk/interpro/entry/InterPro/IPR041118/), [LRR_dom_sf](https://www.ebi.ac.uk/interpro/entry/InterPro/IPR032675/), [Leu-rich_rpt](https://www.ebi.ac.uk/interpro/entry/InterPro/IPR001611/), [WH-like_DNA-bd_sf](https://www.ebi.ac.uk/interpro/entry/InterPro/IPR036388/), [Leu-rich_rpt_typical-subtyp](https://www.ebi.ac.uk/interpro/entry/InterPro/IPR003591/), [P-loop_NTPase](https://www.ebi.ac.uk/interpro/entry/InterPro/IPR027417/), [NB-ARC](https://www.ebi.ac.uk/interpro/entry/InterPro/IPR002182/), [Apaf_helical](https://www.ebi.ac.uk/interpro/entry/InterPro/IPR042197/), [Disease_R_plants](https://www.ebi.ac.uk/interpro/entry/InterPro/IPR044974/) |
| Mi_A_CNL33 | NB-ARC, Rx_N, LRR_8 | Rx_N, NB-ARC | NB-ARC superfamily, RX-CC_like, LRR superfamily, | [WH-like_DNA-bd_sf](https://www.ebi.ac.uk/interpro/entry/InterPro/IPR036388/), [P-loop_NTPase](https://www.ebi.ac.uk/interpro/entry/InterPro/IPR027417/), [LRR_dom_sf](https://www.ebi.ac.uk/interpro/entry/InterPro/IPR032675/), [Rx_N](https://www.ebi.ac.uk/interpro/entry/InterPro/IPR041118/),  [Disease_R_plants](https://www.ebi.ac.uk/interpro/entry/InterPro/IPR044974/), [Apaf_helical](https://www.ebi.ac.uk/interpro/entry/InterPro/IPR042197/), [RX-like_CC](https://www.ebi.ac.uk/interpro/entry/InterPro/IPR038005/), NB-ARC |
| Mi_A_CNL34 | NB-ARC, Rx_N, LRR_8 | Rx_N, NB-ARC | NB-ARC superfamily, RX-CC_like, PLN03210 superfamily | [LRR_dom_sf](https://www.ebi.ac.uk/interpro/entry/InterPro/IPR032675/), [P-loop_NTPase](https://www.ebi.ac.uk/interpro/entry/InterPro/IPR027417/), [NB-ARC](https://www.ebi.ac.uk/interpro/entry/InterPro/IPR002182/), [WH-like_DNA-bd_sf](https://www.ebi.ac.uk/interpro/entry/InterPro/IPR036388/), [Disease_R_plants](https://www.ebi.ac.uk/interpro/entry/InterPro/IPR044974/), [RX-like_CC](https://www.ebi.ac.uk/interpro/entry/InterPro/IPR038005/), [Apaf_helical](https://www.ebi.ac.uk/interpro/entry/InterPro/IPR042197/), [Rx_N](https://www.ebi.ac.uk/interpro/entry/InterPro/IPR041118/) |
| Mi_A_CNL35 | NB-ARC, LRR_8 | Rx_N, NB-ARC | NB-ARC, Rx_N, PLN03210 superfamily, PLN00113 superfamily | [Apaf_helical](https://www.ebi.ac.uk/interpro/entry/InterPro/IPR042197/), [WH-like_DNA-bd_sf](https://www.ebi.ac.uk/interpro/entry/InterPro/IPR036388/), [P-loop_NTPase](https://www.ebi.ac.uk/interpro/entry/InterPro/IPR027417/), RX-like_CC, [LRR_dom_sf](https://www.ebi.ac.uk/interpro/entry/InterPro/IPR032675/), [Rx_N](https://www.ebi.ac.uk/interpro/entry/InterPro/IPR041118/), [Disease_R_plants](https://www.ebi.ac.uk/interpro/entry/InterPro/IPR044974/), [NB-ARC](https://www.ebi.ac.uk/interpro/entry/InterPro/IPR002182/) |
| Mi_A_CNL36 | NB-ARC, Rx_N, LRR_8 | RPW8, NB-ARC | RPW8 superfamily, NB-ARC superfamily, LRR | [RPW8-like](https://www.ebi.ac.uk/interpro/entry/InterPro/IPR039203/), [WH-like_DNA-bd_sf](https://www.ebi.ac.uk/interpro/entry/InterPro/IPR036388/), [Apaf_helical](https://www.ebi.ac.uk/interpro/entry/InterPro/IPR042197/), [NB-ARC](https://www.ebi.ac.uk/interpro/entry/InterPro/IPR002182/), [LRR_dom_sf](https://www.ebi.ac.uk/interpro/entry/InterPro/IPR032675/), [Powdery_mildew-R_dom](https://www.ebi.ac.uk/interpro/entry/InterPro/IPR008808/), [P-loop_NTPase](https://www.ebi.ac.uk/interpro/entry/InterPro/IPR027417/) |
| Mi_A_CNL37 | NB-ARC, Rx_N, LRR_8 | RPW8, NB-ARC | RPW8 superfamily, NB-ARC superfamily, LRR | [NB-ARC](https://www.ebi.ac.uk/interpro/entry/InterPro/IPR002182/), [P-loop_NTPase](https://www.ebi.ac.uk/interpro/entry/InterPro/IPR027417/), [Powdery_mildew-R_dom](https://www.ebi.ac.uk/interpro/entry/InterPro/IPR008808/), [AAA+_ATPase](https://www.ebi.ac.uk/interpro/entry/InterPro/IPR003593/), [WH-like_DNA-bd_sf](https://www.ebi.ac.uk/interpro/entry/InterPro/IPR036388/), [Apaf_helical](https://www.ebi.ac.uk/interpro/entry/InterPro/IPR042197/), [LRR_dom_sf](https://www.ebi.ac.uk/interpro/entry/InterPro/IPR032675/), [RPW8-like](https://www.ebi.ac.uk/interpro/entry/InterPro/IPR039203/) |
| Mi_A_CNL38 | NB-ARC, Rx_N, LRR_8 | Rx_N, NB-ARC | NB-ARC superfamily, RX-CC_like, PLN00113 superfamily | [P-loop_NTPase](https://www.ebi.ac.uk/interpro/entry/InterPro/IPR027417/), [Rx_N](https://www.ebi.ac.uk/interpro/entry/InterPro/IPR041118/), [WH-like_DNA-bd_sf](https://www.ebi.ac.uk/interpro/entry/InterPro/IPR036388/), [RX-like_CC](https://www.ebi.ac.uk/interpro/entry/InterPro/IPR038005/), [Apaf_helical](https://www.ebi.ac.uk/interpro/entry/InterPro/IPR042197/), [Disease_R_plants](https://www.ebi.ac.uk/interpro/entry/InterPro/IPR044974/), [NB-ARC](https://www.ebi.ac.uk/interpro/entry/InterPro/IPR002182/), [LRR_dom_sf](https://www.ebi.ac.uk/interpro/entry/InterPro/IPR032675/) |
| Mi_A_CNL39 | NB-ARC, Rx_N | Rx_N, NB-ARC | NB-ARC superfamily, RX-CC_like, PLN00113 superfamily, | [Apaf_helical](https://www.ebi.ac.uk/interpro/entry/InterPro/IPR042197/), [Rx_N](https://www.ebi.ac.uk/interpro/entry/InterPro/IPR041118/), [P-loop_NTPase](https://www.ebi.ac.uk/interpro/entry/InterPro/IPR027417/), [Disease_R_plants](https://www.ebi.ac.uk/interpro/entry/InterPro/IPR044974/), [RX-like_CC](https://www.ebi.ac.uk/interpro/entry/InterPro/IPR038005/), [LRR_dom_sf](https://www.ebi.ac.uk/interpro/entry/InterPro/IPR032675/), [WH-like_DNA-bd_sf](https://www.ebi.ac.uk/interpro/entry/InterPro/IPR036388/), [NB-ARC](https://www.ebi.ac.uk/interpro/entry/InterPro/IPR002182/) |
| Mi_A_CNL40 | NB-ARC, Rx_N, LRR_8 | Rx_N, NB-ARC, LRR_8 | NB-ARC superfamily, Rx_N, PLN03210 superfamily, PLN03210 superfamily | [LRR_dom_sf](https://www.ebi.ac.uk/interpro/entry/InterPro/IPR032675/), [WH-like_DNA-bd_sf](https://www.ebi.ac.uk/interpro/entry/InterPro/IPR036388/), [NB-ARC](https://www.ebi.ac.uk/interpro/entry/InterPro/IPR002182/), [Leu-rich_rpt](https://www.ebi.ac.uk/interpro/entry/InterPro/IPR001611/), [P-loop_NTPase](https://www.ebi.ac.uk/interpro/entry/InterPro/IPR027417/), [Rx_N](https://www.ebi.ac.uk/interpro/entry/InterPro/IPR041118/), [Apaf_helical](https://www.ebi.ac.uk/interpro/entry/InterPro/IPR042197/), [Disease_R_plants](https://www.ebi.ac.uk/interpro/entry/InterPro/IPR044974/) |
| Mi_A_CNL41 | NB-ARC, Rx_N, LRR_4 | Rx_N, NB-ARC, LRR_8 | NB-ARC superfamily, Rx_N, PLN03210 superfamily, LRR superfamily | [Leu-rich_rpt](https://www.ebi.ac.uk/interpro/entry/InterPro/IPR001611/), [P-loop_NTPase](https://www.ebi.ac.uk/interpro/entry/InterPro/IPR027417/), [P-loop_NTPase](https://www.ebi.ac.uk/interpro/entry/InterPro/IPR027417/), [NB-ARC](https://www.ebi.ac.uk/interpro/entry/InterPro/IPR002182/), [Apaf_helical](https://www.ebi.ac.uk/interpro/entry/InterPro/IPR042197/), [Disease_R_plants](https://www.ebi.ac.uk/interpro/entry/InterPro/IPR044974/), [Rx_N](https://www.ebi.ac.uk/interpro/entry/InterPro/IPR041118/), [LRR_dom_sf](https://www.ebi.ac.uk/interpro/entry/InterPro/IPR032675/) |
| Mi_A_CNL42 | RPW8, NB-ARC | Rx_N, NB-ARC | NB-ARC superfamily, RX-CC_like, LRR | [LRR_dom_sf](https://www.ebi.ac.uk/interpro/entry/InterPro/IPR032675/), [WH-like_DNA-bd_sf](https://www.ebi.ac.uk/interpro/entry/InterPro/IPR036388/), [Apaf_helical](https://www.ebi.ac.uk/interpro/entry/InterPro/IPR042197/), [P-loop_NTPase](https://www.ebi.ac.uk/interpro/entry/InterPro/IPR027417/), [RX-like_CC](https://www.ebi.ac.uk/interpro/entry/InterPro/IPR038005/), [NB-ARC](https://www.ebi.ac.uk/interpro/entry/InterPro/IPR002182/), [Rx_N](https://www.ebi.ac.uk/interpro/entry/InterPro/IPR041118/), [Disease_R_plants](https://www.ebi.ac.uk/interpro/entry/InterPro/IPR044974/) |
| Mi_A_CNL43 | RPW8, NB-ARC | Rx_N, NB-ARC | NB-ARC superfamily, RX-CC_like, LRR | [LRR_dom_sf](https://www.ebi.ac.uk/interpro/entry/InterPro/IPR032675/), [Rx_N](https://www.ebi.ac.uk/interpro/entry/InterPro/IPR041118/), [P-loop_NTPase](https://www.ebi.ac.uk/interpro/entry/InterPro/IPR027417/), [NB-ARC](https://www.ebi.ac.uk/interpro/entry/InterPro/IPR002182/), [Apaf_helical](https://www.ebi.ac.uk/interpro/entry/InterPro/IPR042197/), [Disease_R_plants](https://www.ebi.ac.uk/interpro/entry/InterPro/IPR044974/), [RX-like_CC](https://www.ebi.ac.uk/interpro/entry/InterPro/IPR038005/), [WH-like_DNA-bd_sf](https://www.ebi.ac.uk/interpro/entry/InterPro/IPR036388/) |
| Mi_A_CNL44 | NB-ARC, Rx_N, LRR_8 | Rx_N, NB-ARC | NB-ARC superfamily, RX-CC_like, LRR | [LRR_dom_sf](https://www.ebi.ac.uk/interpro/entry/InterPro/IPR032675/), [Apaf_helical](https://www.ebi.ac.uk/interpro/entry/InterPro/IPR042197/), [Rx_N](https://www.ebi.ac.uk/interpro/entry/InterPro/IPR041118/), [NB-ARC](https://www.ebi.ac.uk/interpro/entry/InterPro/IPR002182/), [Disease_R_plants](https://www.ebi.ac.uk/interpro/entry/InterPro/IPR044974/), [RX-like_CC](https://www.ebi.ac.uk/interpro/entry/InterPro/IPR038005/), [WH-like_DNA-bd_sf](https://www.ebi.ac.uk/interpro/entry/InterPro/IPR036388/), [P-loop_NTPase](https://www.ebi.ac.uk/interpro/entry/InterPro/IPR027417/) |
| Mi_A_CNL45 | NB-ARC, Rx_N, LRR_8 | NB-ARC, LRR_8 | NB-ARC, LRR_8, LRR superfamily | [Leu-rich_rpt](https://www.ebi.ac.uk/interpro/entry/InterPro/IPR001611/), [WH-like_DNA-bd_sf](https://www.ebi.ac.uk/interpro/entry/InterPro/IPR036388/), [Apaf_helical](https://www.ebi.ac.uk/interpro/entry/InterPro/IPR042197/), [Leu-rich_rpt_typical-subtyp](https://www.ebi.ac.uk/interpro/entry/InterPro/IPR003591/), [NB-ARC](https://www.ebi.ac.uk/interpro/entry/InterPro/IPR002182/), [LRR_dom_sf](https://www.ebi.ac.uk/interpro/entry/InterPro/IPR032675/), [P-loop_NTPase](https://www.ebi.ac.uk/interpro/entry/InterPro/IPR027417/) |
| Mi_A_CNL46 | NB-ARC, LRR_8 | Rx_N, NB-ARC | NB-ARC superfamily, RX-CC_like | [LRR_dom_sf](https://www.ebi.ac.uk/interpro/entry/InterPro/IPR032675/), [P-loop_NTPase](https://www.ebi.ac.uk/interpro/entry/InterPro/IPR027417/), [Rx_N](https://www.ebi.ac.uk/interpro/entry/InterPro/IPR041118/), [Disease_R_plants](https://www.ebi.ac.uk/interpro/entry/InterPro/IPR044974/), [Apaf_helical](https://www.ebi.ac.uk/interpro/entry/InterPro/IPR042197/), [WH-like_DNA-bd_sf](https://www.ebi.ac.uk/interpro/entry/InterPro/IPR036388/), [RX-like_CC](https://www.ebi.ac.uk/interpro/entry/InterPro/IPR038005/), [NB-ARC](https://www.ebi.ac.uk/interpro/entry/InterPro/IPR002182/) |
| Mi_A_CNL47 | NB-ARC, Rx_N, LRR_8 | Rx_N, NB-ARC | NB-ARC superfamily, RX-CC_like, LRR superfamily | [P-loop_NTPase](https://www.ebi.ac.uk/interpro/entry/InterPro/IPR027417/), [Apaf_helical](https://www.ebi.ac.uk/interpro/entry/InterPro/IPR042197/), [WH-like_DNA-bd_sf](https://www.ebi.ac.uk/interpro/entry/InterPro/IPR036388/), [NB-ARC](https://www.ebi.ac.uk/interpro/entry/InterPro/IPR002182/), [LRR_dom_sf](https://www.ebi.ac.uk/interpro/entry/InterPro/IPR032675/), [Rx_N](https://www.ebi.ac.uk/interpro/entry/InterPro/IPR041118/), [Disease_R_plants](https://www.ebi.ac.uk/interpro/entry/InterPro/IPR044974/), [RX-like_CC](https://www.ebi.ac.uk/interpro/entry/InterPro/IPR038005/) |
| Mi_H_CNL1 | NB-ARC, Rx_N, LRR_8 | NB-ARC, LRR_8 | NB-ARC superfamily, PLN03210 superfamily, PLN03210 superfamily | [LRR_dom_sf](https://www.ebi.ac.uk/interpro/entry/InterPro/IPR032675/), [Leu-rich_rpt](https://www.ebi.ac.uk/interpro/entry/InterPro/IPR001611/), [Apaf_helical](https://www.ebi.ac.uk/interpro/entry/InterPro/IPR042197/), [Disease_R_plants](https://www.ebi.ac.uk/interpro/entry/InterPro/IPR044974/), [P-loop_NTPase](https://www.ebi.ac.uk/interpro/entry/InterPro/IPR027417/), [NB-ARC](https://www.ebi.ac.uk/interpro/entry/InterPro/IPR002182/), [WH-like_DNA-bd_sf](https://www.ebi.ac.uk/interpro/entry/InterPro/IPR036388/) |
| Mi_H_CNL2 | NB-ARC, Rx_N, LRR_8 | NB-ARC, NB-ARC, LRR_8 | NB-ARC superfamily, NB-ARC superfamily, LRR_8, LRR superfamily, Rx_N, AMN1 superfamily | [P-loop_NTPase](https://www.ebi.ac.uk/interpro/entry/InterPro/IPR027417/), [Leu-rich_rpt_typical-subtyp](https://www.ebi.ac.uk/interpro/entry/InterPro/IPR003591/), [Leu-rich_rpt](https://www.ebi.ac.uk/interpro/entry/InterPro/IPR001611/), [LRR_dom_sf](https://www.ebi.ac.uk/interpro/entry/InterPro/IPR032675/), [WH-like_DNA-bd_sf](https://www.ebi.ac.uk/interpro/entry/InterPro/IPR036388/), [NB-ARC](https://www.ebi.ac.uk/interpro/entry/InterPro/IPR002182/), [Apaf_helical](https://www.ebi.ac.uk/interpro/entry/InterPro/IPR042197/) |
| Mi_H_CNL3 | NB-ARC, Rx_N | NB-ARC | NB-ARC superfamily, PLN00113 superfamily | [P-loop_NTPase](https://www.ebi.ac.uk/interpro/entry/InterPro/IPR027417/), [Apaf_helical](https://www.ebi.ac.uk/interpro/entry/InterPro/IPR042197/), [LRR_dom_sf](https://www.ebi.ac.uk/interpro/entry/InterPro/IPR032675/), [Disease_R_plants](https://www.ebi.ac.uk/interpro/entry/InterPro/IPR044974/), [NB-ARC](https://www.ebi.ac.uk/interpro/entry/InterPro/IPR002182/), [WH-like_DNA-bd_sf](https://www.ebi.ac.uk/interpro/entry/InterPro/IPR036388/) |
| Mi_H_CNL4 | NB-ARC, Rx_N, LRR_8 | NB-ARC, NB-ARC, LRR_8 | NB-ARC superfamily, NB-ARC superfamily, LRR_8, PLN00113 superfamily, bZIP superfamily | [LRR_dom_sf](https://www.ebi.ac.uk/interpro/entry/InterPro/IPR032675/), [P-loop_NTPase](https://www.ebi.ac.uk/interpro/entry/InterPro/IPR027417/), [Leu-rich_rpt](https://www.ebi.ac.uk/interpro/entry/InterPro/IPR001611/), [Apaf_helical](https://www.ebi.ac.uk/interpro/entry/InterPro/IPR042197/), [NB-ARC](https://www.ebi.ac.uk/interpro/entry/InterPro/IPR002182/) |
| Mi_H_CNL5 | NB-ARC, Rx_N, LRR_4 | NB-ARC, LRR_8 | NB-ARC superfamily, LRR | [P-loop_NTPase](https://www.ebi.ac.uk/interpro/entry/InterPro/IPR027417/), [NB-ARC](https://www.ebi.ac.uk/interpro/entry/InterPro/IPR002182/), [Leu-rich_rpt](https://www.ebi.ac.uk/interpro/entry/InterPro/IPR001611/), [LRR_dom_sf](https://www.ebi.ac.uk/interpro/entry/InterPro/IPR032675/), [Apaf_helical](https://www.ebi.ac.uk/interpro/entry/InterPro/IPR042197/) |
| Mi_H_CNL6 | RPW8, NB-ARC | NB-ARC, LRR_8 | NB-ARC superfamily, LRR | [P-loop_NTPase](https://www.ebi.ac.uk/interpro/entry/InterPro/IPR027417/), [NB-ARC](https://www.ebi.ac.uk/interpro/entry/InterPro/IPR002182/), [LRR_dom_sf](https://www.ebi.ac.uk/interpro/entry/InterPro/IPR032675/), [Apaf_helical](https://www.ebi.ac.uk/interpro/entry/InterPro/IPR042197/), [WH-like_DNA-bd_sf](https://www.ebi.ac.uk/interpro/entry/InterPro/IPR036388/), [Leu-rich_rpt_typical-subtyp](https://www.ebi.ac.uk/interpro/entry/InterPro/IPR003591/), [Leu-rich_rpt](https://www.ebi.ac.uk/interpro/entry/InterPro/IPR001611/) |
| Mi_H_CNL7 | RPW8, NB-ARC | Rx_N, NB-ARC | NB-ARC, RX-CC_like, PLN03210 superfamily, PLN00113 superfamily | [Disease_R_plants](https://www.ebi.ac.uk/interpro/entry/InterPro/IPR044974/), [P-loop_NTPase](https://www.ebi.ac.uk/interpro/entry/InterPro/IPR027417/), [RX-like_CC](https://www.ebi.ac.uk/interpro/entry/InterPro/IPR038005/), [NB-ARC](https://www.ebi.ac.uk/interpro/entry/InterPro/IPR002182/), [WH-like_DNA-bd_sf](https://www.ebi.ac.uk/interpro/entry/InterPro/IPR036388/), [Apaf_helical](https://www.ebi.ac.uk/interpro/entry/InterPro/IPR042197/), [LRR_dom_sf](https://www.ebi.ac.uk/interpro/entry/InterPro/IPR032675/), [Rx_N](https://www.ebi.ac.uk/interpro/entry/InterPro/IPR041118/) |
| Mi_H_CNL8 | NB-ARC, Rx_N, LRR_8 | RPW8, NB-ARC, Pkinase | PKc_like superfamily, RPW8 superfamily, NB-ARC superfamily, LRR superfamily | [Prot_kinase_dom](https://www.ebi.ac.uk/interpro/entry/InterPro/IPR000719/), [NB-ARC](https://www.ebi.ac.uk/interpro/entry/InterPro/IPR002182/), [Protein_kinase_ATP_BS](https://www.ebi.ac.uk/interpro/entry/InterPro/IPR017441/), [Powdery_mildew-R_dom](https://www.ebi.ac.uk/interpro/entry/InterPro/IPR008808/), [WH-like_DNA-bd_sf](https://www.ebi.ac.uk/interpro/entry/InterPro/IPR036388/), [RPW8-like](https://www.ebi.ac.uk/interpro/entry/InterPro/IPR039203/), [Apaf_helical](https://www.ebi.ac.uk/interpro/entry/InterPro/IPR042197/), [P-loop_NTPase](https://www.ebi.ac.uk/interpro/entry/InterPro/IPR027417/), [LRR_dom_sf](https://www.ebi.ac.uk/interpro/entry/InterPro/IPR032675/), [Kinase-like_dom_sf](https://www.ebi.ac.uk/interpro/entry/InterPro/IPR011009/) |
| Mi_H_CNL9 | NB-ARC, Rx_N, LRR_8 | RPW8, NB-ARC | RPW8 superfamily, NB-ARC superfamily, LRR | [WH-like_DNA-bd_sf](https://www.ebi.ac.uk/interpro/entry/InterPro/IPR036388/), [Apaf_helical](https://www.ebi.ac.uk/interpro/entry/InterPro/IPR042197/), [NB-ARC](https://www.ebi.ac.uk/interpro/entry/InterPro/IPR002182/), [P-loop_NTPase](https://www.ebi.ac.uk/interpro/entry/InterPro/IPR027417/), [Powdery_mildew-R_dom](https://www.ebi.ac.uk/interpro/entry/InterPro/IPR008808/), [LRR_dom_sf](https://www.ebi.ac.uk/interpro/entry/InterPro/IPR032675/), [RPW8-like](https://www.ebi.ac.uk/interpro/entry/InterPro/IPR039203/) |
| Mi_H_CNL10 | NB-ARC, LRR_8 | Rx_N, NB-ARC | NB-ARC superfamily, RX-CC_like, LRR | [Disease_R_plants](https://www.ebi.ac.uk/interpro/entry/InterPro/IPR044974/), [P-loop_NTPase](https://www.ebi.ac.uk/interpro/entry/InterPro/IPR027417/), [LRR_dom_sf](https://www.ebi.ac.uk/interpro/entry/InterPro/IPR032675/), [NB-ARC](https://www.ebi.ac.uk/interpro/entry/InterPro/IPR002182/), [RX-like_CC](https://www.ebi.ac.uk/interpro/entry/InterPro/IPR038005/), [Apaf_helical](https://www.ebi.ac.uk/interpro/entry/InterPro/IPR042197/), [Rx_N](https://www.ebi.ac.uk/interpro/entry/InterPro/IPR041118/) |
| Mi_H_CNL11 | NB-ARC, Rx_N, LRR_8 | Rx_N, NB-ARC | NB-ARC superfamily, RX-CC_like, PLN03210 superfamily | [WH-like_DNA-bd_sf](https://www.ebi.ac.uk/interpro/entry/InterPro/IPR036388/), [P-loop_NTPase](https://www.ebi.ac.uk/interpro/entry/InterPro/IPR027417/), [NB-ARC](https://www.ebi.ac.uk/interpro/entry/InterPro/IPR002182/), [Disease_R_plants](https://www.ebi.ac.uk/interpro/entry/InterPro/IPR044974/), [Rx_N](https://www.ebi.ac.uk/interpro/entry/InterPro/IPR041118/), [Apaf_helical](https://www.ebi.ac.uk/interpro/entry/InterPro/IPR042197/), [RX-like_CC](https://www.ebi.ac.uk/interpro/entry/InterPro/IPR038005/), [LRR_dom_sf](https://www.ebi.ac.uk/interpro/entry/InterPro/IPR032675/) |
| Mi_H_CNL12 | NB-ARC, Rx_N, LRR_8 | NB-ARC | NB-ARC superfamily, PLN00113 superfamily | [NB-ARC](https://www.ebi.ac.uk/interpro/entry/InterPro/IPR002182/), [Disease_R_plants](https://www.ebi.ac.uk/interpro/entry/InterPro/IPR044974/), [P-loop_NTPase](https://www.ebi.ac.uk/interpro/entry/InterPro/IPR027417/), [LRR_dom_sf](https://www.ebi.ac.uk/interpro/entry/InterPro/IPR032675/), |
| Mi_H_CNL13 | NB-ARC, Rx_N, LRR_8 | NB-ARC | NB-ARC superfamily, PLN00113 superfamily | [LRR_dom_sf](https://www.ebi.ac.uk/interpro/entry/InterPro/IPR032675/), [P-loop_NTPase](https://www.ebi.ac.uk/interpro/entry/InterPro/IPR027417/), [NB-ARC](https://www.ebi.ac.uk/interpro/entry/InterPro/IPR002182/), [WH-like_DNA-bd_sf](https://www.ebi.ac.uk/interpro/entry/InterPro/IPR036388/), [Disease_R_plants](https://www.ebi.ac.uk/interpro/entry/InterPro/IPR044974/) |
| Mi_H_CNL14 | NB-ARC, Rx_N | NB-ARC, LRR_8 | NB-ARC superfamily, LRR superfamily, PLN03210 superfamily | [NB-ARC](https://www.ebi.ac.uk/interpro/entry/InterPro/IPR002182/), [P-loop_NTPase](https://www.ebi.ac.uk/interpro/entry/InterPro/IPR027417/), [Apaf_helical](https://www.ebi.ac.uk/interpro/entry/InterPro/IPR042197/), [LRR_dom_sf](https://www.ebi.ac.uk/interpro/entry/InterPro/IPR032675/), [Leu-rich_rpt](https://www.ebi.ac.uk/interpro/entry/InterPro/IPR001611/) |
| Mi_H_CNL15 | NB-ARC, Rx_N, LRR_8 | NB-ARC, LRR_8 | NB-ARC, LRR_8, LRR superfamily, PLN03210 superfamily | [LRR_dom_sf](https://www.ebi.ac.uk/interpro/entry/InterPro/IPR032675/), [P-loop_NTPase](https://www.ebi.ac.uk/interpro/entry/InterPro/IPR027417/), [NB-ARC](https://www.ebi.ac.uk/interpro/entry/InterPro/IPR002182/), [Leu-rich_rpt](https://www.ebi.ac.uk/interpro/entry/InterPro/IPR001611/), [WH-like_DNA-bd_sf](https://www.ebi.ac.uk/interpro/entry/InterPro/IPR036388/), [Leu-rich_rpt_typical-subtyp](https://www.ebi.ac.uk/interpro/entry/InterPro/IPR003591/), [Apaf_helical](https://www.ebi.ac.uk/interpro/entry/InterPro/IPR042197/) |
| Mi_H_CNL16 | NB-ARC, Rx_N, LRR_4 | Rx_N, NB-ARC | NB-ARC superfamily, RX-CC_like, LRR | [Rx_N](https://www.ebi.ac.uk/interpro/entry/InterPro/IPR041118/), [WH-like_DNA-bd_sf](https://www.ebi.ac.uk/interpro/entry/InterPro/IPR036388/), [RX-like_CC](https://www.ebi.ac.uk/interpro/entry/InterPro/IPR038005/), [P-loop_NTPase](https://www.ebi.ac.uk/interpro/entry/InterPro/IPR027417/), [NB-ARC](https://www.ebi.ac.uk/interpro/entry/InterPro/IPR002182/), [Disease_R_plants](https://www.ebi.ac.uk/interpro/entry/InterPro/IPR044974/), [Apaf_helical](https://www.ebi.ac.uk/interpro/entry/InterPro/IPR042197/), [LRR_dom_sf](https://www.ebi.ac.uk/interpro/entry/InterPro/IPR032675/) |
| Mi_H_CNL17 | RPW8, NB-ARC | Rx_N, NB-ARC | NB-ARC superfamily, RX-CC_like, PLN03210 superfamily | [P-loop_NTPase](https://www.ebi.ac.uk/interpro/entry/InterPro/IPR027417/), [RX-like_CC](https://www.ebi.ac.uk/interpro/entry/InterPro/IPR038005/), [LRR_dom_sf](https://www.ebi.ac.uk/interpro/entry/InterPro/IPR032675/), [Apaf_helical](https://www.ebi.ac.uk/interpro/entry/InterPro/IPR042197/), [Disease_R_plants](https://www.ebi.ac.uk/interpro/entry/InterPro/IPR044974/), [Rx_N](https://www.ebi.ac.uk/interpro/entry/InterPro/IPR041118/), [NB-ARC](https://www.ebi.ac.uk/interpro/entry/InterPro/IPR002182/) |
| Mi_H_CNL18 | RPW8, NB-ARC | NB-ARC, Ank_2, Ank_2, LRR_8 | NB-ARC superfamily, Ank_2, NB-ARC superfamily, LRR | [Leu-rich_rpt_typical-subtyp](https://www.ebi.ac.uk/interpro/entry/InterPro/IPR003591/), [Ankyrin_rpt](https://www.ebi.ac.uk/interpro/entry/InterPro/IPR002110/), [P-loop_NTPase](https://www.ebi.ac.uk/interpro/entry/InterPro/IPR027417/), [LRR_dom_sf](https://www.ebi.ac.uk/interpro/entry/InterPro/IPR032675/), [Ankyrin_rpt-contain_sf](https://www.ebi.ac.uk/interpro/entry/InterPro/IPR036770/), [Apaf_helical](https://www.ebi.ac.uk/interpro/entry/InterPro/IPR042197/), [NB-ARC](https://www.ebi.ac.uk/interpro/entry/InterPro/IPR002182/), [Leu-rich_rpt](https://www.ebi.ac.uk/interpro/entry/InterPro/IPR001611/), [WH-like_DNA-bd_sf](https://www.ebi.ac.uk/interpro/entry/InterPro/IPR036388/) |
| Mi_H_CNL19 | NB-ARC, Rx_N, LRR_8 | NB-ARC, Ank_2, Ank_2, LRR_8 | NB-ARC superfamily, Ank_2, NB-ARC superfamily, PPP1R42 superfamily | [Apaf_helical](https://www.ebi.ac.uk/interpro/entry/InterPro/IPR042197/), [Ankyrin_rpt](https://www.ebi.ac.uk/interpro/entry/InterPro/IPR002110/), [Ankyrin_rpt-contain_sf](https://www.ebi.ac.uk/interpro/entry/InterPro/IPR036770/), [P-loop_NTPase](https://www.ebi.ac.uk/interpro/entry/InterPro/IPR027417/), [NB-ARC](https://www.ebi.ac.uk/interpro/entry/InterPro/IPR002182/), [WH-like_DNA-bd_sf](https://www.ebi.ac.uk/interpro/entry/InterPro/IPR036388/), [LRR_dom_sf](https://www.ebi.ac.uk/interpro/entry/InterPro/IPR032675/), [Leu-rich_rpt](https://www.ebi.ac.uk/interpro/entry/InterPro/IPR001611/), [Leu-rich_rpt_typical-subtyp](https://www.ebi.ac.uk/interpro/entry/InterPro/IPR003591/) |
| Mi_H_CNL20 | NB-ARC, Rx_N, LRR_8 | NB-ARC, Ank_2, Ank_2, LRR_8 | NB-ARC superfamily, Ank_2, NB-ARC superfamily, LRR | [NB-ARC](https://www.ebi.ac.uk/interpro/entry/InterPro/IPR002182/), [Ankyrin_rpt](https://www.ebi.ac.uk/interpro/entry/InterPro/IPR002110/), [P-loop_NTPase](https://www.ebi.ac.uk/interpro/entry/InterPro/IPR027417/), [Leu-rich_rpt_typical-subtyp](https://www.ebi.ac.uk/interpro/entry/InterPro/IPR003591/), [LRR_dom_sf](https://www.ebi.ac.uk/interpro/entry/InterPro/IPR032675/), [Leu-rich_rpt](https://www.ebi.ac.uk/interpro/entry/InterPro/IPR001611/), [Ankyrin_rpt-contain_sf](https://www.ebi.ac.uk/interpro/entry/InterPro/IPR036770/), [WH-like_DNA-bd_sf](https://www.ebi.ac.uk/interpro/entry/InterPro/IPR036388/), [Apaf_helical](https://www.ebi.ac.uk/interpro/entry/InterPro/IPR042197/) |
| Mi_H_CNL21 | NB-ARC, LRR_8 | RPW8, NB-ARC | NB-ARC superfamily, RPW8 superfamily | [Powdery_mildew-R_dom](https://www.ebi.ac.uk/interpro/entry/InterPro/IPR008808/), [Apaf_helical](https://www.ebi.ac.uk/interpro/entry/InterPro/IPR042197/), [P-loop_NTPase](https://www.ebi.ac.uk/interpro/entry/InterPro/IPR027417/), [RPW8-like](https://www.ebi.ac.uk/interpro/entry/InterPro/IPR039203/), [NB-ARC](https://www.ebi.ac.uk/interpro/entry/InterPro/IPR002182/), [WH-like_DNA-bd_sf](https://www.ebi.ac.uk/interpro/entry/InterPro/IPR036388/) |
| Mi_H_CNL22 | NB-ARC, Rx_N, LRR_8 | RPW8, NB-ARC | NB-ARC superfamily, RPW8 superfamily, PLN03210 superfamily | [P-loop_NTPase](https://www.ebi.ac.uk/interpro/entry/InterPro/IPR027417/), [RPW8-like](https://www.ebi.ac.uk/interpro/entry/InterPro/IPR039203/), [Apaf_helical](https://www.ebi.ac.uk/interpro/entry/InterPro/IPR042197/), [NB-ARC](https://www.ebi.ac.uk/interpro/entry/InterPro/IPR002182/), [Powdery_mildew-R_dom](https://www.ebi.ac.uk/interpro/entry/InterPro/IPR008808/), [WH-like_DNA-bd_sf](https://www.ebi.ac.uk/interpro/entry/InterPro/IPR036388/), [LRR_dom_sf](https://www.ebi.ac.uk/interpro/entry/InterPro/IPR032675/) |
| Mi_H_CNL23 | NB-ARC, Rx_N, LRR_8 | Rx_N, NB-ARC | NB-ARC superfamily, Rx_N | [P-loop_NTPase](https://www.ebi.ac.uk/interpro/entry/InterPro/IPR027417/), [Disease_R_plants](https://www.ebi.ac.uk/interpro/entry/InterPro/IPR044974/), [NB-ARC](https://www.ebi.ac.uk/interpro/entry/InterPro/IPR002182/), [Rx_N](https://www.ebi.ac.uk/interpro/entry/InterPro/IPR041118/) |
| Mi_H_CNL24 | NB-ARC, Rx_N, LRR_8 | NB-ARC | NB-ARC superfamily, PLN03210 superfamily | [P-loop_NTPase](https://www.ebi.ac.uk/interpro/entry/InterPro/IPR027417/), [LRR_dom_sf](https://www.ebi.ac.uk/interpro/entry/InterPro/IPR032675/), [Apaf_helical](https://www.ebi.ac.uk/interpro/entry/InterPro/IPR042197/), [Disease_R_plants](https://www.ebi.ac.uk/interpro/entry/InterPro/IPR044974/), [NB-ARC](https://www.ebi.ac.uk/interpro/entry/InterPro/IPR002182/), [WH-like_DNA-bd_sf](https://www.ebi.ac.uk/interpro/entry/InterPro/IPR036388/) |
| Mi_H_CNL25 | NB-ARC, Rx_N | RPW8, NB-ARC | RPW8 superfamily, NB-ARC superfamily, PLN03210 superfamily, LRR superfamily | [Powdery_mildew-R_dom](https://www.ebi.ac.uk/interpro/entry/InterPro/IPR008808/), [WH-like_DNA-bd_sf](https://www.ebi.ac.uk/interpro/entry/InterPro/IPR036388/), [Apaf_helical](https://www.ebi.ac.uk/interpro/entry/InterPro/IPR042197/), [RPW8-like](https://www.ebi.ac.uk/interpro/entry/InterPro/IPR039203/), [P-loop_NTPase](https://www.ebi.ac.uk/interpro/entry/InterPro/IPR027417/), [LRR_dom_sf](https://www.ebi.ac.uk/interpro/entry/InterPro/IPR032675/), [NB-ARC](https://www.ebi.ac.uk/interpro/entry/InterPro/IPR002182/) |
| Mi_H_CNL26 | NB-ARC, Rx_N, LRR_8 | NB-ARC, LRR_8 | NB-ARC superfamily, LRR superfamily | [Leu-rich_rpt_typical-subtyp](https://www.ebi.ac.uk/interpro/entry/InterPro/IPR003591/), [Leu-rich_rpt](https://www.ebi.ac.uk/interpro/entry/InterPro/IPR001611/), [LRR_dom_sf](https://www.ebi.ac.uk/interpro/entry/InterPro/IPR032675/), [NB-ARC](https://www.ebi.ac.uk/interpro/entry/InterPro/IPR002182/), [P-loop_NTPase](https://www.ebi.ac.uk/interpro/entry/InterPro/IPR027417/), [Apaf_helical](https://www.ebi.ac.uk/interpro/entry/InterPro/IPR042197/), [WH-like_DNA-bd_sf](https://www.ebi.ac.uk/interpro/entry/InterPro/IPR036388/) |
| Mi_H_CNL27 | NB-ARC, Rx_N, LRR_4 | Rx_N, NB-ARC | NB-ARC superfamily, RX-CC_like, LRR superfamily | [P-loop_NTPase](https://www.ebi.ac.uk/interpro/entry/InterPro/IPR027417/), [Rx_N](https://www.ebi.ac.uk/interpro/entry/InterPro/IPR041118/), [Disease_R_plants](https://www.ebi.ac.uk/interpro/entry/InterPro/IPR044974/), [WH-like_DNA-bd_sf](https://www.ebi.ac.uk/interpro/entry/InterPro/IPR036388/), [LRR_dom_sf](https://www.ebi.ac.uk/interpro/entry/InterPro/IPR032675/), [Apaf_helical](https://www.ebi.ac.uk/interpro/entry/InterPro/IPR042197/), [NB-ARC](https://www.ebi.ac.uk/interpro/entry/InterPro/IPR002182/), [RX-like_CC](https://www.ebi.ac.uk/interpro/entry/InterPro/IPR038005/) |
| Mi_T_CNL1 | RPW8, NB-ARC | Rx_N, NB-ARC, LRR_8 | NB-ARC superfamily, Rx_N, PLN03210 superfamily, PLN03210 superfamily | [LRR_dom_sf](https://www.ebi.ac.uk/interpro/entry/InterPro/IPR032675/), [Rx_N](https://www.ebi.ac.uk/interpro/entry/InterPro/IPR041118/), [P-loop_NTPase](https://www.ebi.ac.uk/interpro/entry/InterPro/IPR027417/), [Disease_R_plants](https://www.ebi.ac.uk/interpro/entry/InterPro/IPR044974/), [Leu-rich_rpt](https://www.ebi.ac.uk/interpro/entry/InterPro/IPR001611/), [RX-like_CC](https://www.ebi.ac.uk/interpro/entry/InterPro/IPR038005/), [NB-ARC](https://www.ebi.ac.uk/interpro/entry/InterPro/IPR002182/), [WH-like_DNA-bd_sf](https://www.ebi.ac.uk/interpro/entry/InterPro/IPR036388/), [Apaf_helical](https://www.ebi.ac.uk/interpro/entry/InterPro/IPR042197/) |
| Mi_T_CNL2 | RPW8, NB-ARC | Rx_N, NB-ARC | NB-ARC superfamily, RX-CC_like, PLN00113 superfamily | [RX-like_CC](https://www.ebi.ac.uk/interpro/entry/InterPro/IPR038005/), [NB-ARC](https://www.ebi.ac.uk/interpro/entry/InterPro/IPR002182/), [Rx_N](https://www.ebi.ac.uk/interpro/entry/InterPro/IPR041118/), [WH-like_DNA-bd_sf](https://www.ebi.ac.uk/interpro/entry/InterPro/IPR036388/), [LRR_dom_sf](https://www.ebi.ac.uk/interpro/entry/InterPro/IPR032675/), [Disease_R_plants](https://www.ebi.ac.uk/interpro/entry/InterPro/IPR044974/), [P-loop_NTPase](https://www.ebi.ac.uk/interpro/entry/InterPro/IPR027417/), [Apaf_helical](https://www.ebi.ac.uk/interpro/entry/InterPro/IPR042197/) |
| Mi_T_CNL3 | NB-ARC, Rx_N, LRR_8 | Rx_N, NB-ARC | NB-ARC superfamily, RX-CC_like | [P-loop_NTPase](https://www.ebi.ac.uk/interpro/entry/InterPro/IPR027417/), [RX-like_CC](https://www.ebi.ac.uk/interpro/entry/InterPro/IPR038005/), [Rx_N](https://www.ebi.ac.uk/interpro/entry/InterPro/IPR041118/), [Apaf_helical](https://www.ebi.ac.uk/interpro/entry/InterPro/IPR042197/), [Disease_R_plants](https://www.ebi.ac.uk/interpro/entry/InterPro/IPR044974/), [LRR_dom_sf](https://www.ebi.ac.uk/interpro/entry/InterPro/IPR032675/), [NB-ARC](https://www.ebi.ac.uk/interpro/entry/InterPro/IPR002182/), [WH-like_DNA-bd_sf](https://www.ebi.ac.uk/interpro/entry/InterPro/IPR036388/) |
| Mi_T_CNL4 | NB-ARC, Rx_N, LRR_8 | Rx_N, NB-ARC, Rx_N, NB-ARC | NB-ARC superfamily, NB-ARC superfamily, RX-CC_like, RX-CC_like, LRR superfamily, LRR superfamily | [P-loop_NTPase](https://www.ebi.ac.uk/interpro/entry/InterPro/IPR027417/), [Disease_R_plants](https://www.ebi.ac.uk/interpro/entry/InterPro/IPR044974/), [LRR_dom_sf](https://www.ebi.ac.uk/interpro/entry/InterPro/IPR032675/), [WH-like_DNA-bd_sf](https://www.ebi.ac.uk/interpro/entry/InterPro/IPR036388/), [Apaf_helical](https://www.ebi.ac.uk/interpro/entry/InterPro/IPR042197/), [RX-like_CC](https://www.ebi.ac.uk/interpro/entry/InterPro/IPR038005/), [AAA+_ATPase](https://www.ebi.ac.uk/interpro/entry/InterPro/IPR003593/), [NB-ARC](https://www.ebi.ac.uk/interpro/entry/InterPro/IPR002182/), [Rx_N](https://www.ebi.ac.uk/interpro/entry/InterPro/IPR041118/) |
| Mi_T_CNL5 | NB-ARC, LRR_8 | Rx_N, NB-ARC | NB-ARC superfamily, RX-CC_like, PLN03210 superfamily | [WH-like_DNA-bd_sf](https://www.ebi.ac.uk/interpro/entry/InterPro/IPR036388/), [NB-ARC](https://www.ebi.ac.uk/interpro/entry/InterPro/IPR002182/), [Rx_N](https://www.ebi.ac.uk/interpro/entry/InterPro/IPR041118/), [RX-like_CC](https://www.ebi.ac.uk/interpro/entry/InterPro/IPR038005/), [Apaf_helical](https://www.ebi.ac.uk/interpro/entry/InterPro/IPR042197/), [P-loop_NTPase](https://www.ebi.ac.uk/interpro/entry/InterPro/IPR027417/), [LRR_dom_sf](https://www.ebi.ac.uk/interpro/entry/InterPro/IPR032675/), [Disease_R_plants](https://www.ebi.ac.uk/interpro/entry/InterPro/IPR044974/) |
| Mi_T_CNL6 | NB-ARC, Rx_N, LRR_8 | NB-ARC, LRR_8 | NB-ARC superfamily, LRR_8, LRR superfamily | [P-loop_NTPase](https://www.ebi.ac.uk/interpro/entry/InterPro/IPR027417/), [Leu-rich_rpt_typical-subtyp](https://www.ebi.ac.uk/interpro/entry/InterPro/IPR003591/), [Apaf_helical](https://www.ebi.ac.uk/interpro/entry/InterPro/IPR042197/), [LRR_dom_sf](https://www.ebi.ac.uk/interpro/entry/InterPro/IPR032675/), [Leu-rich_rpt](https://www.ebi.ac.uk/interpro/entry/InterPro/IPR001611/), [WH-like_DNA-bd_sf](https://www.ebi.ac.uk/interpro/entry/InterPro/IPR036388/), [NB-ARC](https://www.ebi.ac.uk/interpro/entry/InterPro/IPR002182/) |
| Mi_T_CNL7 | NB-ARC, Rx_N, LRR_8 | NB-ARC, LRR_8, NB-ARC, LRR_8 | NB-ARC, NB-ARC, LRR_8, LRR, PLN00113 superfamily, bZIP superfamily | [LRR_dom_sf](https://www.ebi.ac.uk/interpro/entry/InterPro/IPR032675/), [P-loop_NTPase](https://www.ebi.ac.uk/interpro/entry/InterPro/IPR027417/), [Leu-rich_rpt](https://www.ebi.ac.uk/interpro/entry/InterPro/IPR001611/), [Apaf_helical](https://www.ebi.ac.uk/interpro/entry/InterPro/IPR042197/), [Leu-rich_rpt_typical-subtyp](https://www.ebi.ac.uk/interpro/entry/InterPro/IPR003591/), [AAA+_ATPase](https://www.ebi.ac.uk/interpro/entry/InterPro/IPR003593/), [NB-ARC](https://www.ebi.ac.uk/interpro/entry/InterPro/IPR002182/), [WH-like_DNA-bd_sf](https://www.ebi.ac.uk/interpro/entry/InterPro/IPR036388/) |
| Mi_T_CNL8 | NB-ARC, Rx_N, LRR_8 | NB-ARC, LRR_8 | NB-ARC superfamily, LRR | [LRR_dom_sf](https://www.ebi.ac.uk/interpro/entry/InterPro/IPR032675/), [Leu-rich_rpt](https://www.ebi.ac.uk/interpro/entry/InterPro/IPR001611/), [P-loop_NTPase](https://www.ebi.ac.uk/interpro/entry/InterPro/IPR027417/), [WH-like_DNA-bd_sf](https://www.ebi.ac.uk/interpro/entry/InterPro/IPR036388/), [NB-ARC](https://www.ebi.ac.uk/interpro/entry/InterPro/IPR002182/), [Apaf_helical](https://www.ebi.ac.uk/interpro/entry/InterPro/IPR042197/) |
| Mi_T_CNL9 | NB-ARC, Rx_N | Rx_N, NB-ARC, CRAL_TRIO | NB-ARC, SEC14, Rx_N, PLN03210 superfamily, PLN00113 superfamily | [CRAL-TRIO_dom](https://www.ebi.ac.uk/interpro/entry/InterPro/IPR001251/), [LRR_dom_sf](https://www.ebi.ac.uk/interpro/entry/InterPro/IPR032675/), [WH-like_DNA-bd_sf](https://www.ebi.ac.uk/interpro/entry/InterPro/IPR036388/), [CRAL-TRIO_dom_sf](https://www.ebi.ac.uk/interpro/entry/InterPro/IPR036865/), [NB-ARC](https://www.ebi.ac.uk/interpro/entry/InterPro/IPR002182/), [Apaf_helical](https://www.ebi.ac.uk/interpro/entry/InterPro/IPR042197/), [P-loop_NTPase](https://www.ebi.ac.uk/interpro/entry/InterPro/IPR027417/), [Rx_N](https://www.ebi.ac.uk/interpro/entry/InterPro/IPR041118/), [RX-like_CC](https://www.ebi.ac.uk/interpro/entry/InterPro/IPR038005/), [CRAL/TRIO_N_dom_sf](https://www.ebi.ac.uk/interpro/entry/InterPro/IPR036273/) |
| Mi_T_CNL10 | NB-ARC, Rx_N, LRR_8 | Rx_N, NB-ARC | NB-ARC superfamily, RX-CC_like, LRR | [NB-ARC](https://www.ebi.ac.uk/interpro/entry/InterPro/IPR002182/), [P-loop_NTPase](https://www.ebi.ac.uk/interpro/entry/InterPro/IPR027417/), [Apaf_helical](https://www.ebi.ac.uk/interpro/entry/InterPro/IPR042197/), [LRR_dom_sf](https://www.ebi.ac.uk/interpro/entry/InterPro/IPR032675/), [RX-like_CC](https://www.ebi.ac.uk/interpro/entry/InterPro/IPR038005/), [Rx_N](https://www.ebi.ac.uk/interpro/entry/InterPro/IPR041118/), [Disease_R_plants](https://www.ebi.ac.uk/interpro/entry/InterPro/IPR044974/), [WH-like_DNA-bd_sf](https://www.ebi.ac.uk/interpro/entry/InterPro/IPR036388/) |
| Mi_T_CNL11 | NB-ARC, Rx_N, LRR_4 | Rx_N, NB-ARC | NB-ARC superfamily, RX-CC_like, LRR | [WH-like_DNA-bd_sf](https://www.ebi.ac.uk/interpro/entry/InterPro/IPR036388/), [LRR_dom_sf](https://www.ebi.ac.uk/interpro/entry/InterPro/IPR032675/), [RX-like_CC](https://www.ebi.ac.uk/interpro/entry/InterPro/IPR038005/), [P-loop_NTPase](https://www.ebi.ac.uk/interpro/entry/InterPro/IPR027417/), [NB-ARC](https://www.ebi.ac.uk/interpro/entry/InterPro/IPR002182/), [Apaf_helical](https://www.ebi.ac.uk/interpro/entry/InterPro/IPR042197/), [Rx_N](https://www.ebi.ac.uk/interpro/entry/InterPro/IPR041118/), [Disease_R_plants](https://www.ebi.ac.uk/interpro/entry/InterPro/IPR044974/) |
| Mi_T_CNL12 | RPW8, NB-ARC | NB-ARC | NB-ARC superfamily, PLN03210 superfamily, PLN03210 superfamily | [P-loop_NTPase](https://www.ebi.ac.uk/interpro/entry/InterPro/IPR027417/), [NB-ARC](https://www.ebi.ac.uk/interpro/entry/InterPro/IPR002182/), [LRR_dom_sf](https://www.ebi.ac.uk/interpro/entry/InterPro/IPR032675/), [Apaf_helical](https://www.ebi.ac.uk/interpro/entry/InterPro/IPR042197/), [Disease_R_plants](https://www.ebi.ac.uk/interpro/entry/InterPro/IPR044974/), [WH-like_DNA-bd_sf](https://www.ebi.ac.uk/interpro/entry/InterPro/IPR036388/) |
| Mi_T_CNL13 | RPW8, NB-ARC | NB-ARC, LRR_8 | NB-ARC superfamily, LRR | [Leu-rich_rpt](https://www.ebi.ac.uk/interpro/entry/InterPro/IPR001611/), [NB-ARC](https://www.ebi.ac.uk/interpro/entry/InterPro/IPR002182/), [LRR_dom_sf](https://www.ebi.ac.uk/interpro/entry/InterPro/IPR032675/), [WH-like_DNA-bd_sf](https://www.ebi.ac.uk/interpro/entry/InterPro/IPR036388/), [P-loop_NTPase](https://www.ebi.ac.uk/interpro/entry/InterPro/IPR027417/), [Apaf_helical](https://www.ebi.ac.uk/interpro/entry/InterPro/IPR042197/) |
| Mi_T_CNL14 | NB-ARC, Rx_N, LRR_8 | NB-ARC, LRR_8 | NB-ARC superfamily, LRR superfamily, PLN03210 superfamily | [P-loop_NTPase](https://www.ebi.ac.uk/interpro/entry/InterPro/IPR027417/), [NB-ARC](https://www.ebi.ac.uk/interpro/entry/InterPro/IPR002182/), [LRR_dom_sf](https://www.ebi.ac.uk/interpro/entry/InterPro/IPR032675/), [Apaf_helical](https://www.ebi.ac.uk/interpro/entry/InterPro/IPR042197/), [Leu-rich_rpt](https://www.ebi.ac.uk/interpro/entry/InterPro/IPR001611/) |
| Mi_T_CNL15 | NB-ARC, Rx_N, LRR_8 | NB-ARC, LRR_8 | NB-ARC superfamily, LRR_8, PLN03210 superfamily | [Leu-rich_rpt](https://www.ebi.ac.uk/interpro/entry/InterPro/IPR001611/), [LRR_dom_sf](https://www.ebi.ac.uk/interpro/entry/InterPro/IPR032675/), [P-loop_NTPase](https://www.ebi.ac.uk/interpro/entry/InterPro/IPR027417/), [NB-ARC](https://www.ebi.ac.uk/interpro/entry/InterPro/IPR002182/), [Leu-rich_rpt_typical-subtyp](https://www.ebi.ac.uk/interpro/entry/InterPro/IPR003591/) |
| Mi_T_CNL16 | NB-ARC, LRR_8 | Rx_N, NB-ARC | NB-ARC superfamily, RX-CC_like, LRR | [WH-like_DNA-bd_sf](https://www.ebi.ac.uk/interpro/entry/InterPro/IPR036388/), [RX-like_CC](https://www.ebi.ac.uk/interpro/entry/InterPro/IPR038005/), [NB-ARC](https://www.ebi.ac.uk/interpro/entry/InterPro/IPR002182/), [P-loop_NTPase](https://www.ebi.ac.uk/interpro/entry/InterPro/IPR027417/), [Apaf_helical](https://www.ebi.ac.uk/interpro/entry/InterPro/IPR042197/), [LRR_dom_sf](https://www.ebi.ac.uk/interpro/entry/InterPro/IPR032675/), [Rx_N](https://www.ebi.ac.uk/interpro/entry/InterPro/IPR041118/), [Disease_R_plants](https://www.ebi.ac.uk/interpro/entry/InterPro/IPR044974/) |
| Mi_T_CNL17 | NB-ARC, Rx_N, LRR_8 | Rx_N, NB-ARC | NB-ARC superfamily, RX-CC_like, LRR | [P-loop_NTPase](https://www.ebi.ac.uk/interpro/entry/InterPro/IPR027417/), [RX-like_CC](https://www.ebi.ac.uk/interpro/entry/InterPro/IPR038005/), [Apaf_helical](https://www.ebi.ac.uk/interpro/entry/InterPro/IPR042197/), [NB-ARC](https://www.ebi.ac.uk/interpro/entry/InterPro/IPR002182/), [Rx_N](https://www.ebi.ac.uk/interpro/entry/InterPro/IPR041118/), [LRR_dom_sf](https://www.ebi.ac.uk/interpro/entry/InterPro/IPR032675/), [Disease_R_plants](https://www.ebi.ac.uk/interpro/entry/InterPro/IPR044974/), [WH-like_DNA-bd_sf](https://www.ebi.ac.uk/interpro/entry/InterPro/IPR036388/) |
| Mi_T_CNL18 | NB-ARC, Rx_N, LRR_8 | Rx_N, NB-ARC | NB-ARC superfamily, RX-CC_like, LRR superfamily | [Disease_R_plants](https://www.ebi.ac.uk/interpro/entry/InterPro/IPR044974/), [RX-like_CC](https://www.ebi.ac.uk/interpro/entry/InterPro/IPR038005/), [Apaf_helical](https://www.ebi.ac.uk/interpro/entry/InterPro/IPR042197/), [P-loop_NTPase](https://www.ebi.ac.uk/interpro/entry/InterPro/IPR027417/), [LRR_dom_sf](https://www.ebi.ac.uk/interpro/entry/InterPro/IPR032675/), [Rx_N](https://www.ebi.ac.uk/interpro/entry/InterPro/IPR041118/), [WH-like_DNA-bd_sf](https://www.ebi.ac.uk/interpro/entry/InterPro/IPR036388/), [NB-ARC](https://www.ebi.ac.uk/interpro/entry/InterPro/IPR002182/) |
| Mi_T_CNL19 | NB-ARC, Rx_N, LRR_8 | NB-ARC, Rx_N, NB-ARC | NB-ARC superfamily, RX-CC_like, NB-ARC superfamily, PLN03210 superfamily, LRR superfamily | [LRR_dom_sf](https://www.ebi.ac.uk/interpro/entry/InterPro/IPR032675/), [NB-ARC](https://www.ebi.ac.uk/interpro/entry/InterPro/IPR002182/), [P-loop_NTPase](https://www.ebi.ac.uk/interpro/entry/InterPro/IPR027417/), [Disease_R_plants](https://www.ebi.ac.uk/interpro/entry/InterPro/IPR044974/), [Rx_N](https://www.ebi.ac.uk/interpro/entry/InterPro/IPR041118/), [Leu-rich_rpt_typical-subtyp](https://www.ebi.ac.uk/interpro/entry/InterPro/IPR003591/), [WH-like_DNA-bd_sf](https://www.ebi.ac.uk/interpro/entry/InterPro/IPR036388/), [Apaf_helical](https://www.ebi.ac.uk/interpro/entry/InterPro/IPR042197/), [RX-like_CC](https://www.ebi.ac.uk/interpro/entry/InterPro/IPR038005/) |
| Mi_T_CNL20 | NB-ARC, Rx_N | Rx_N, NB-ARC, LRR_8 | NB-ARC superfamily, Rx_N, PLN03210 superfamily | [Leu-rich_rpt](https://www.ebi.ac.uk/interpro/entry/InterPro/IPR001611/), [P-loop_NTPase](https://www.ebi.ac.uk/interpro/entry/InterPro/IPR027417/), [NB-ARC](https://www.ebi.ac.uk/interpro/entry/InterPro/IPR002182/), [Rx_N](https://www.ebi.ac.uk/interpro/entry/InterPro/IPR041118/), [LRR_dom_sf](https://www.ebi.ac.uk/interpro/entry/InterPro/IPR032675/), [RX-like_CC](https://www.ebi.ac.uk/interpro/entry/InterPro/IPR038005/), [Disease_R_plants](https://www.ebi.ac.uk/interpro/entry/InterPro/IPR044974/), [WH-like_DNA-bd_sf](https://www.ebi.ac.uk/interpro/entry/InterPro/IPR036388/) |
| Mi_T_CNL21 | NB-ARC, Rx_N, LRR_8 | RPW8, NB-ARC, RPW8, NB-ARC | NB-ARC superfamily, NB-ARC superfamily, PLN03210 superfamily, PLN03210 superfamily, RPW8 superfamily | [P-loop_NTPase](https://www.ebi.ac.uk/interpro/entry/InterPro/IPR027417/), [Apaf_helical](https://www.ebi.ac.uk/interpro/entry/InterPro/IPR042197/), [Powdery_mildew-R_dom](https://www.ebi.ac.uk/interpro/entry/InterPro/IPR008808/), [WH-like_DNA-bd_sf](https://www.ebi.ac.uk/interpro/entry/InterPro/IPR036388/), [LRR_dom_sf](https://www.ebi.ac.uk/interpro/entry/InterPro/IPR032675/), [RPW8-like](https://www.ebi.ac.uk/interpro/entry/InterPro/IPR039203/), [NB-ARC](https://www.ebi.ac.uk/interpro/entry/InterPro/IPR002182/), [AAA+_ATPase](https://www.ebi.ac.uk/interpro/entry/InterPro/IPR003593/) |
| Mi_T_CNL22 | NB-ARC, Rx_N, LRR_4 | RPW8, NB-ARC | NB-ARC superfamily, RPW8 superfamily | [Powdery_mildew-R_dom](https://www.ebi.ac.uk/interpro/entry/InterPro/IPR008808/), [Adaptor_Cbl_N_dom_sf](https://www.ebi.ac.uk/interpro/entry/InterPro/IPR036537/), [RPW8-like](https://www.ebi.ac.uk/interpro/entry/InterPro/IPR039203/), [WH-like_DNA-bd_sf](https://www.ebi.ac.uk/interpro/entry/InterPro/IPR036388/), [NB-ARC](https://www.ebi.ac.uk/interpro/entry/InterPro/IPR002182/), [Apaf_helical](https://www.ebi.ac.uk/interpro/entry/InterPro/IPR042197/), [P-loop_NTPase](https://www.ebi.ac.uk/interpro/entry/InterPro/IPR027417/) |
| Mi_T_CNL23 | RPW8, NB-ARC | Rx_N, NB-ARC, CaM_binding | NB-ARC superfamily, CaM_binding, Rx_N, PLN03210 superfamily, PLN03200 superfamily, PLN00113 superfamily, PLN03210 superfamily | [P-loop_NTPase](https://www.ebi.ac.uk/interpro/entry/InterPro/IPR027417/), [Apaf_helical](https://www.ebi.ac.uk/interpro/entry/InterPro/IPR042197/), [Rx_N](https://www.ebi.ac.uk/interpro/entry/InterPro/IPR041118/), [CaM-bd_dom_pln](https://www.ebi.ac.uk/interpro/entry/InterPro/IPR012417/), [ARM-like](https://www.ebi.ac.uk/interpro/entry/InterPro/IPR011989/), [LRR_dom_sf](https://www.ebi.ac.uk/interpro/entry/InterPro/IPR032675/), [ARM-type_fold](https://www.ebi.ac.uk/interpro/entry/InterPro/IPR016024/), [Armadillo](https://www.ebi.ac.uk/interpro/entry/InterPro/IPR000225/), [WH-like_DNA-bd_sf](https://www.ebi.ac.uk/interpro/entry/InterPro/IPR036388/), [Leu-rich_rpt_typical-subtyp](https://www.ebi.ac.uk/interpro/entry/InterPro/IPR003591/), [NB-ARC](https://www.ebi.ac.uk/interpro/entry/InterPro/IPR002182/) |
| Mi_T_CNL24 | RPW8, NB-ARC | Rx_N, NB-ARC | NB-ARC superfamily, RX-CC_like | [RX-like_CC](https://www.ebi.ac.uk/interpro/entry/InterPro/IPR038005/), [WH-like_DNA-bd_sf](https://www.ebi.ac.uk/interpro/entry/InterPro/IPR036388/), [LRR_dom_sf](https://www.ebi.ac.uk/interpro/entry/InterPro/IPR032675/), [P-loop_NTPase](https://www.ebi.ac.uk/interpro/entry/InterPro/IPR027417/), [NB-ARC](https://www.ebi.ac.uk/interpro/entry/InterPro/IPR002182/), [Apaf_helical](https://www.ebi.ac.uk/interpro/entry/InterPro/IPR042197/), [Disease_R_plants](https://www.ebi.ac.uk/interpro/entry/InterPro/IPR044974/), [Rx_N](https://www.ebi.ac.uk/interpro/entry/InterPro/IPR041118/) |
| Mi_T_CNL25 | NB-ARC, Rx_N, LRR_8 | Rx_N, NB-ARC, Alg14, Rx_N, Rx_N, NB-ARC, Alg14, Alg14 | NB-ARC superfamily, NB-ARC superfamily, Glycosyltransferase_GTB-type superfamily, Rx_N, PLN03210 superfamily | [WH-like_DNA-bd_sf](https://www.ebi.ac.uk/interpro/entry/InterPro/IPR036388/), [Oligosacch_biosynth_Alg14](https://www.ebi.ac.uk/interpro/entry/InterPro/IPR013969/), [P-loop_NTPase](https://www.ebi.ac.uk/interpro/entry/InterPro/IPR027417/), [Disease_R_plants](https://www.ebi.ac.uk/interpro/entry/InterPro/IPR044974/), [LRR_dom_sf](https://www.ebi.ac.uk/interpro/entry/InterPro/IPR032675/), [Leu-rich_rpt_typical-subtyp](https://www.ebi.ac.uk/interpro/entry/InterPro/IPR003591/), [NB-ARC](https://www.ebi.ac.uk/interpro/entry/InterPro/IPR002182/), [RX-like_CC](https://www.ebi.ac.uk/interpro/entry/InterPro/IPR038005/), [Rx_N](https://www.ebi.ac.uk/interpro/entry/InterPro/IPR041118/), [Apaf_helical](https://www.ebi.ac.uk/interpro/entry/InterPro/IPR042197/), [Leu-rich_rpt_Cys-con_subtyp](https://www.ebi.ac.uk/interpro/entry/InterPro/IPR006553/) |
| Mi_T_CNL26 | NB-ARC, Rx_N, LRR_8 | Rx_N, NB-ARC | NB-ARC superfamily, RX-CC_like, PLN00113 superfamily | [RX-like_CC](https://www.ebi.ac.uk/interpro/entry/InterPro/IPR038005/), [WH-like_DNA-bd_sf](https://www.ebi.ac.uk/interpro/entry/InterPro/IPR036388/), [NB-ARC](https://www.ebi.ac.uk/interpro/entry/InterPro/IPR002182/), [P-loop_NTPase](https://www.ebi.ac.uk/interpro/entry/InterPro/IPR027417/), [Disease_R_plants](https://www.ebi.ac.uk/interpro/entry/InterPro/IPR044974/), [LRR_dom_sf](https://www.ebi.ac.uk/interpro/entry/InterPro/IPR032675/), [Rx_N](https://www.ebi.ac.uk/interpro/entry/InterPro/IPR041118/), [Apaf_helical](https://www.ebi.ac.uk/interpro/entry/InterPro/IPR042197/) |
| Mi_T_CNL27 | NB-ARC, LRR_8 | RPW8, NB-ARC | RPW8 superfamily, NB-ARC superfamily, PLN03210 superfamily, LRR superfamily | [Apaf_helical](https://www.ebi.ac.uk/interpro/entry/InterPro/IPR042197/), [RPW8-like](https://www.ebi.ac.uk/interpro/entry/InterPro/IPR039203/), [P-loop_NTPase](https://www.ebi.ac.uk/interpro/entry/InterPro/IPR027417/), [LRR_dom_sf](https://www.ebi.ac.uk/interpro/entry/InterPro/IPR032675/), [WH-like_DNA-bd_sf](https://www.ebi.ac.uk/interpro/entry/InterPro/IPR036388/), [Powdery_mildew-R_dom](https://www.ebi.ac.uk/interpro/entry/InterPro/IPR008808/), [NB-ARC](https://www.ebi.ac.uk/interpro/entry/InterPro/IPR002182/) |
| Mi_T_CNL28 | NB-ARC, Rx_N, LRR_8 | Rx_N, NB-ARC | NB-ARC superfamily, RX-CC_like, PLN03210 superfamily | [RX-like_CC](https://www.ebi.ac.uk/interpro/entry/InterPro/IPR038005/), [LRR_dom_sf](https://www.ebi.ac.uk/interpro/entry/InterPro/IPR032675/), [NB-ARC](https://www.ebi.ac.uk/interpro/entry/InterPro/IPR002182/), [Disease_R_plants](https://www.ebi.ac.uk/interpro/entry/InterPro/IPR044974/), [Rx_N](https://www.ebi.ac.uk/interpro/entry/InterPro/IPR041118/), [P-loop_NTPase](https://www.ebi.ac.uk/interpro/entry/InterPro/IPR027417/), [Apaf_helical](https://www.ebi.ac.uk/interpro/entry/InterPro/IPR042197/), [WH-like_DNA-bd_sf](https://www.ebi.ac.uk/interpro/entry/InterPro/IPR036388/) |
| Mi_T_CNL29 | NB-ARC, Rx_N, LRR_8 | Rx_N, NB-ARC | NB-ARC superfamily, RX-CC_like, PLN00113 superfamily | [Disease_R_plants](https://www.ebi.ac.uk/interpro/entry/InterPro/IPR044974/), [P-loop_NTPase](https://www.ebi.ac.uk/interpro/entry/InterPro/IPR027417/), [RX-like_CC](https://www.ebi.ac.uk/interpro/entry/InterPro/IPR038005/), [LRR_dom_sf](https://www.ebi.ac.uk/interpro/entry/InterPro/IPR032675/), [WH-like_DNA-bd_sf](https://www.ebi.ac.uk/interpro/entry/InterPro/IPR036388/), [NB-ARC](https://www.ebi.ac.uk/interpro/entry/InterPro/IPR002182/), [Rx_N](https://www.ebi.ac.uk/interpro/entry/InterPro/IPR041118/) |
| Mi_T_CNL30 | NB-ARC, Rx_N, LRR_8 | Rx_N, NB-ARC | NB-ARC superfamily, Rx_N, LRR | [NB-ARC](https://www.ebi.ac.uk/interpro/entry/InterPro/IPR002182/), [Apaf_helical](https://www.ebi.ac.uk/interpro/entry/InterPro/IPR042197/), [Disease_R_plants](https://www.ebi.ac.uk/interpro/entry/InterPro/IPR044974/), [P-loop_NTPase](https://www.ebi.ac.uk/interpro/entry/InterPro/IPR027417/), [Rx_N](https://www.ebi.ac.uk/interpro/entry/InterPro/IPR041118/), [LRR_dom_sf](https://www.ebi.ac.uk/interpro/entry/InterPro/IPR032675/), [RX-like_CC](https://www.ebi.ac.uk/interpro/entry/InterPro/IPR038005/), [WH-like_DNA-bd_sf](https://www.ebi.ac.uk/interpro/entry/InterPro/IPR036388/) |
| Mi_T_CNL31 | NB-ARC, Rx_N | Rx_N, NB-ARC | NB-ARC superfamily, RX-CC_like, PLN00113 superfamily | [RX-like_CC](https://www.ebi.ac.uk/interpro/entry/InterPro/IPR038005/), [WH-like_DNA-bd_sf](https://www.ebi.ac.uk/interpro/entry/InterPro/IPR036388/), [LRR_dom_sf](https://www.ebi.ac.uk/interpro/entry/InterPro/IPR032675/), [P-loop_NTPase](https://www.ebi.ac.uk/interpro/entry/InterPro/IPR027417/), [NB-ARC](https://www.ebi.ac.uk/interpro/entry/InterPro/IPR002182/), [Rx_N](https://www.ebi.ac.uk/interpro/entry/InterPro/IPR041118/), [Disease_R_plants](https://www.ebi.ac.uk/interpro/entry/InterPro/IPR044974/) |
| Mi_T_CNL32 | NB-ARC, Rx_N, LRR_8 | NB-ARC, LRR_8 | NB-ARC superfamily, PLN03210 superfamily | [P-loop_NTPase](https://www.ebi.ac.uk/interpro/entry/InterPro/IPR027417/), [Leu-rich_rpt](https://www.ebi.ac.uk/interpro/entry/InterPro/IPR001611/), [WH-like_DNA-bd_sf](https://www.ebi.ac.uk/interpro/entry/InterPro/IPR036388/), [LRR_dom_sf](https://www.ebi.ac.uk/interpro/entry/InterPro/IPR032675/), [Leu-rich_rpt_typical-subtyp](https://www.ebi.ac.uk/interpro/entry/InterPro/IPR003591/), [NB-ARC](https://www.ebi.ac.uk/interpro/entry/InterPro/IPR002182/), [Apaf_helical](https://www.ebi.ac.uk/interpro/entry/InterPro/IPR042197/) |
| Mi_T_CNL33 | NB-ARC, Rx_N, LRR_4 | NB-ARC, LRR_8 | NB-ARC superfamily, PLN03210 superfamily, LRR superfamily | [LRR_dom_sf](https://www.ebi.ac.uk/interpro/entry/InterPro/IPR032675/), [P-loop_NTPase](https://www.ebi.ac.uk/interpro/entry/InterPro/IPR027417/), [WH-like_DNA-bd_sf](https://www.ebi.ac.uk/interpro/entry/InterPro/IPR036388/), [Apaf_helical](https://www.ebi.ac.uk/interpro/entry/InterPro/IPR042197/), [NB-ARC](https://www.ebi.ac.uk/interpro/entry/InterPro/IPR002182/), [Leu-rich_rpt](https://www.ebi.ac.uk/interpro/entry/InterPro/IPR001611/), [Disease_R_plants](https://www.ebi.ac.uk/interpro/entry/InterPro/IPR044974/) |
| Mi_T_CNL34 | RPW8, NB-ARC | Rx_N, NB-ARC | NB-ARC superfamily, Rx_N, PLN03210 superfamily | [Rx_N](https://www.ebi.ac.uk/interpro/entry/InterPro/IPR041118/), [Apaf_helical](https://www.ebi.ac.uk/interpro/entry/InterPro/IPR042197/), [NB-ARC](https://www.ebi.ac.uk/interpro/entry/InterPro/IPR002182/), [P-loop_NTPase](https://www.ebi.ac.uk/interpro/entry/InterPro/IPR027417/), [RX-like_CC](https://www.ebi.ac.uk/interpro/entry/InterPro/IPR038005/), [Disease_R_plants](https://www.ebi.ac.uk/interpro/entry/InterPro/IPR044974/), [LRR_dom_sf](https://www.ebi.ac.uk/interpro/entry/InterPro/IPR032675/), [WH-like_DNA-bd_sf](https://www.ebi.ac.uk/interpro/entry/InterPro/IPR036388/) |
| Mi_T_CNL35 | RPW8, NB-ARC | NB-ARC, LRR_8 | NB-ARC superfamily, LRR superfamily, PLN03210 superfamily | [LRR_dom_sf](https://www.ebi.ac.uk/interpro/entry/InterPro/IPR032675/), [P-loop_NTPase](https://www.ebi.ac.uk/interpro/entry/InterPro/IPR027417/), [WH-like_DNA-bd_sf](https://www.ebi.ac.uk/interpro/entry/InterPro/IPR036388/), [NB-ARC](https://www.ebi.ac.uk/interpro/entry/InterPro/IPR002182/), [Leu-rich_rpt](https://www.ebi.ac.uk/interpro/entry/InterPro/IPR001611/), [Apaf_helical](https://www.ebi.ac.uk/interpro/entry/InterPro/IPR042197/) |
| Mi_T_CNL36 | NB-ARC, Rx_N, LRR_8 | Rx_N, NB-ARC, Rx_N, NB-ARC | NB-ARC superfamily, RX-CC_like, LRR superfamily | [NB-ARC](https://www.ebi.ac.uk/interpro/entry/InterPro/IPR002182/), [P-loop_NTPase](https://www.ebi.ac.uk/interpro/entry/InterPro/IPR027417/), [LRR_dom_sf](https://www.ebi.ac.uk/interpro/entry/InterPro/IPR032675/), [Apaf_helical](https://www.ebi.ac.uk/interpro/entry/InterPro/IPR042197/), [RX-like_CC](https://www.ebi.ac.uk/interpro/entry/InterPro/IPR038005/), [WH-like_DNA-bd_sf](https://www.ebi.ac.uk/interpro/entry/InterPro/IPR036388/), [Disease_R_plants](https://www.ebi.ac.uk/interpro/entry/InterPro/IPR044974/), [AAA+_ATPase](https://www.ebi.ac.uk/interpro/entry/InterPro/IPR003593/), [Rx_N](https://www.ebi.ac.uk/interpro/entry/InterPro/IPR041118/) |
